# Efficacy and safety of V-Loc^™^ barbed sutures versus conventional suture techniques in gynecological surgery: a systematic review and meta-analysis

**DOI:** 10.1007/s00404-023-07291-3

**Published:** 2023-12-21

**Authors:** Juliane Hafermann, Ubong Silas, Rhodri Saunders

**Affiliations:** Coreva Scientific GmbH & Co KG, Im Muehlenbruch 1, 53639 Koenigswinter, Germany

**Keywords:** V-Loc, Barbed suture, Hysterectomy, Myomectomy, Meta-analysis

## Abstract

**Purpose:**

One of the most challenging tasks in laparoscopic gynecological surgeries is suturing. Knotless barbed sutures are intended to enable faster suturing and hemostasis. We carried out a meta-analysis to compare the efficacy and safety of V-Loc^™^ barbed sutures (VBS) with conventional sutures (CS) in gynecological surgeries.

**Methods:**

We systematically searched PubMed and EMBASE for studies published between 2010 and September 2021 comparing VBS to CS for OB/GYN procedures. All comparative studies were included. Primary analysis and subgroup analyses for the different surgery and suturing types were performed. Primary outcomes were operation time and suture time; secondary outcomes included post-operative complications, surgical site infections, estimated blood loss, length of stay, granulation tissue formation, and surgical difficulty. Results were calculated as weighted mean difference (WMD) or risk ratio (RR) and 95% confidence intervals (CI) with a random effects model, and a sensitivity analysis for study quality, study size, and outlier results was performed. PROSPERO registration: CRD42022363187.

**Results:**

In total, 25 studies involving 4452 women undergoing hysterectomy, myomectomy, or excision of endometrioma. VBS were associated with a reduction in operation time (WMD – 17.08 min; 95% CI – 21.57, – 12.59), suture time (WMD – 5.39 min; 95% CI – 7.06, – 3.71), surgical site infection (RR 0.26; 95% CI 0.09, 0.78), estimated blood loss (WMD – 44.91 ml; 95% CI – 66.01, – 23.81), granulation tissue formation (RR 0.48; 95% CI 0.25, 0.89), and surgical difficulty (WMD – 1.98 VAS score; 95% CI – 2.83, – 1.13). No difference between VBS and CS was found regarding total postoperative complications or length of stay. Many of the outcomes showed high heterogeneity, likely due to the inclusion of different surgery types and comparators. Most results were shown to be robust in the sensitivity analysis except for the reduction in granulation tissue formation.

**Conclusion:**

This meta-analysis indicates that V-Loc^™^ barbed sutures are safe and effective in gynecological surgeries as they reduce operation time, suture time, blood loss, infections, and surgical difficulty without increasing post-operative complications or length of stay compared to conventional sutures.

**Supplementary Information:**

The online version contains supplementary material available at 10.1007/s00404-023-07291-3.

## What does this study add to the clinical work

**Table:** 

This meta-analysis shows that V-Loc^™^ barbed sutures facilitate gynecological surgery. They save time and are associated with reduced blood loss, infections and surgical difficulty compared to conventional sutures.	

## Introduction

Gynecological procedures are frequently performed using minimally invasive techniques [1–3], and incisions in the uterine wall or vaginal vault are often closed with conventional sutures using synthetic material. However, intracorporeal knot-tying is one of the most challenging and time-consuming tasks for laparoscopic surgeons [4–6]. Knotless barbed sutures promising to reduce the difficulties of laparoscopic suturing were first used in gynecologic surgery in 2008 [4, 6].

As barbed sutures avoid the need for knots, they prevent associated weaknesses such as uneven tension, slippage, and knots as focal points for tissue stress which may promote lacerations, necrosis, and infection [4, 6, 7]. Barbed sutures evenly distribute and maintain tension along the wound without the need to readjust suture tension intraoperatively [5, 8, 9]. This equal distribution of tension may support wound healing and prevent localized hypoxia and inflammation without compromising the strength of the suture [6].

Different kinds of barbed sutures are available, such as absorbable versus non-absorbable or unidirectional versus bidirectional barbs [4, 6]. V-Loc^™^ barbed sutures (VBS) by Medtronic are a range of monofilament unidirectional barbed sutures available in different sizes, absorption properties, and tensile strength. V-Loc^™^ 90 and V-Loc^™^ 180 are absorbable sutures with an absorption profile of 90 days and 180 days, respectively, while V-Loc PBT is non-absorbable. VBS have dual-angle cut barbs that anchor the suture in the tissue, provide tissue approximation, and prevent slippage without requiring knots [5]. They have been used in a wide range of procedures, including urologic [10], gastrointestinal [11], orthopedic [12], and plastic surgeries [13].

## Objectives

Many studies that analyzed the effectiveness of VBS in obstetric and gynecological (OB/GYN) procedures have small patient populations and only few are randomized clinical trials. Furthermore, previous meta-analyses on the use of barbed sutures in OB/GYN surgery pooled the results [14–18] despite differences between the different types of barbed sutures [19–21].

Therefore, we performed a systematic review and meta-analysis of all comparative studies comparing VBS to conventional sutures (CS) with the aim to provide a more accurate assessment of the efficacy and safety of VBS in OB/GYN surgery.

## Methods

The protocol for this systematic review and meta-analysis was registered with PROSPERO (registration number CRD42022363187). This analysis did not deviate from the protocol. The study findings were reported according to the PRISMA checklist [22].

### Search strategy

We searched PubMed and EMBASE on 6 September 2021 for relevant studies. The searches were restricted to studies on human patients published between 2010 (VBS were initially launched in October 2009) and September 2021 in English, French, and German language. An effort was made to identify all relevant published and peer-reviewed comparative studies addressing the safety and efficacy of VBS in OB/GYN surgeries.

The search strategy identified studies that used Medical Subject Headings, Emtree terms, and free text terms related to “V-Loc”, “barbed suture” or “knotless suture” or “self-retaining suture”, and excluded studies that only used terms related to “Quill” or “Stratafix” (other commercially available barbed sutures). Detailed search strings were published in the PROSPERO protocol. All identified studies and duplicate records were screened using PICO Portal (www.picoportal.org). Studies that passed screening were managed in the Citavi reference management software.

### Eligibility criteria

Studies were selected based on the following inclusion criteria: we included peer-reviewed comparative studies comparing VBS to CS in any tissue layer of OB/GYN surgeries and reporting at least one outcome measure of OB/GYN surgery. The study population included all women undergoing any OB/GYN procedure allocated either to wound closure with VBS (study group) or with CS (control group).

Abstract-only, editorials, commentaries, review articles not presenting original data, single-arm studies, case studies, and case reports were excluded. The initial search included studies on all types of surgery, and non-OB/GYN studies were only excluded during full-text review.

### Study selection

Studies identified in the systematic search were retrieved and screened independently for inclusion by two researchers. Each study’s eligibility was initially assessed based on its title and abstract, followed by a full-text review of the studies that passed abstract screening to determine their inclusion for data extraction and meta-analysis. Researchers were blinded to each other’s decisions. Disagreements between the two researchers regarding the inclusion or exclusion of studies were settled by a third independent reviewer.

### Data extraction

Data were extracted by two independent researchers from the included studies using a pre-piloted Excel sheet. Disagreements were resolved through discussion and consensus between the two researchers, or through discussion with a third reviewer if necessary. The extracted data included study design, location, time frame, population, intervention, comparison, and surgery type as well as participant and intervention characteristics.

Primary outcomes were suture time and operation time in minutes. Secondary outcomes were perioperative blood loss, length of hospital stay, rate of total post-operative complications, surgical site infections, and development of granulation tissue, as well as surgical difficulty rating. All reported results (for all measures, time points, and analyses) were collected for all outcomes.

### Assessment of risk of bias

The risk of bias of included studies was assessed using the modified Downs and Black checklist for quality assessment for both randomized controlled trials (RCTs) and non-RCTs [23]. The domains of assessment included study quality, external validity, study bias, confounding and selection bias, and power of the study. The risk of bias assessment was performed by two independent reviewers. Disagreements were either resolved by discussion among the two reviewers or with an additional third reviewer. Score ranges were grouped into the following four quality levels: excellent (26–28), good (20–25), fair (15–19), and poor (≤ 14) [24].

### Data synthesis

Meta-analyses were performed to assess the overall outcomes of wound closure with VBS compared to CS. Continuous variables were calculated from the means and standard deviations (SD) of each group and synthesized as weighted mean difference (WMD) ± 95% confidence interval (CI). The mean and standard deviation were estimated from median and range or interquartile range using methods described by Wan et al. (2014) [25], where applicable. If only mean and range were reported, the standard deviation was estimated as the range divided by 4. Categorical variables were summarized as risk ratios (RR) ± 95% CI.

#### Primary analysis

Due to the broad inclusion criteria of this review, considerable heterogeneity between studies was anticipated. Therefore, it was decided a priori to perform the meta-analysis using the random effects model. The meta-analysis results are visualized in Forest plots.

We tabulated the study characteristics and the reported results of all studies eligible for data synthesis and ensured that the study matched the selection criteria outlined in the section on study selection. We included all studies in the data syntheses that reported continuous outcomes as mean and standard deviation (or other formats that can be used to estimate the mean and standard deviation as described earlier in this manuscript) and categorical outcomes as numbers of events and/or rates in percent.

Study heterogeneity was evaluated using *I*^2^ values expressed as a percentage for each outcome, with a value ≥ 50% considered an indicator of substantial heterogeneity. A *p* value < 0.05 was considered statistically significant. All statistical analyses and graphical presentations were performed using Microsoft Excel and Review Manager (RevMan), Version 5.4 by The Cochrane Collaboration, 2020.

#### Subgroup analyses

Subgroup analyses were performed primarily for surgical procedures (hysterectomy, myomectomy, and excision of endometrioma), and secondarily for different CS techniques (interrupted and running) to explore possible causes for heterogeneity among study results.

#### Sensitivity analyses

Sensitivity analyses were performed to assess the impact of (a) studies with high risk of bias, (b) disproportionately large studies, and (c) studies reporting extreme outcomes. For this, the meta-analyses were repeated excluding (a) studies rated “poor” risk of bias assessment, (b) the 10% (*n* = 3) studies with the largest number of patients, and (c) studies whose confidence interval does not overlap with the confidence interval of the pooled effect.

The sensitivity analysis results were compared to the original meta-analyses, and the difference between them expressed as a percentage to determine which outcomes were most affected by the factors assessed in the sensitivity analyses and to assess the robustness of the synthesized results.

### Assessment of reporting bias

Funnel plots were generated in RevMan for each outcome. The symmetry of the outcome distribution can be an indication for small study effects, heterogeneity, reporting bias, or publication bias. As the random effects model assumes that the included studies have different true effects, it is not possible to show the 95% CI in the funnel plots.

## Results

**Fig. 1:**
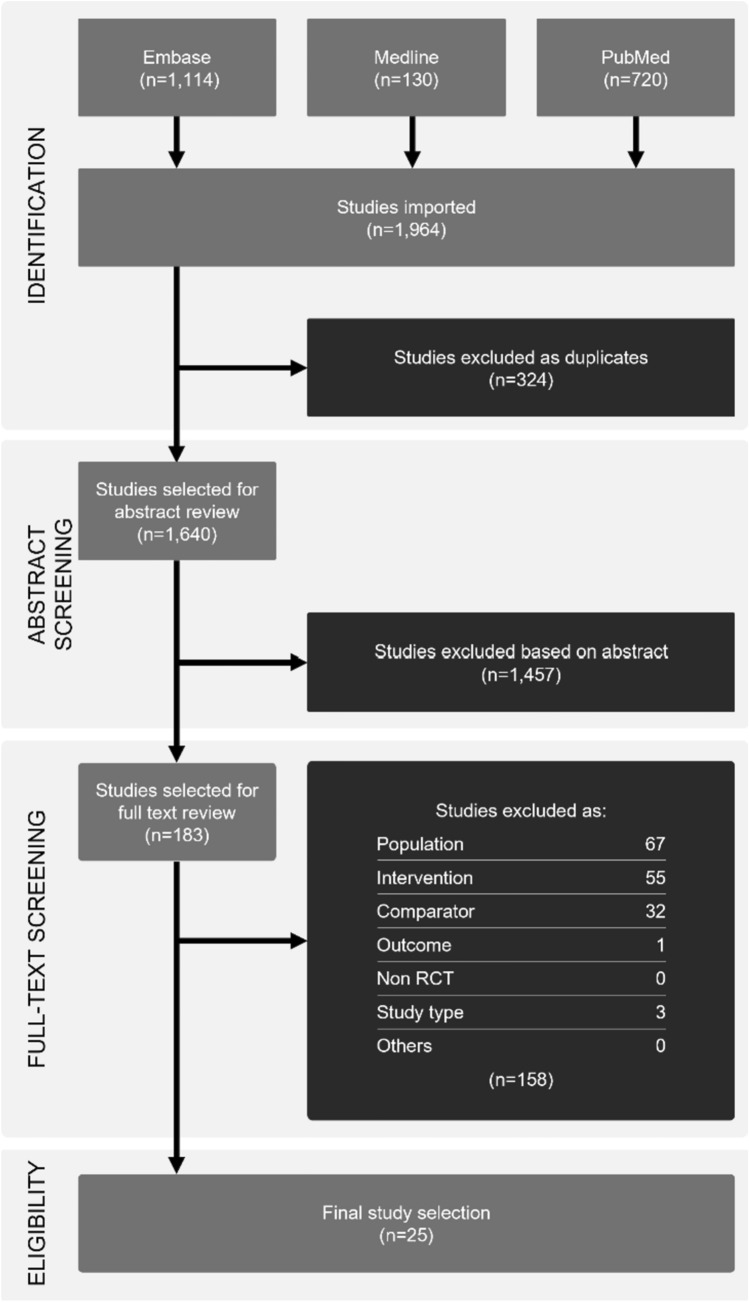
Study selection for this systematic review and meta-analysis outlined in a PRISMA flowchart

Study selection is presented in a PRISMA diagram (Fig. 1).

### Study selection

The initial search resulted in 1640 unique articles, of which 183 were found relevant after abstract and title review. A total of 25 studies met all criteria and were finally included in the meta-analysis (Fig. 1). Combined, these studies included 4452 women (2298 in the study group and 2154 in the control group).

### Study characteristics

The study characteristics are summarized in the supplementary material (Table S1). All included studies described gynecological procedures; no comparative studies on VBS in obstetrics were identified.

Subgroup analysis between the different procedures was performed for the 16 studies on hysterectomy, 8 studies on myomectomy and 1 study on the excision of endometrioma (Table 1) where barbed sutures were used to close the vaginal cuff, the uterine wall, or the endometrioma bed, respectively.

**Table 1:** Outcome measures and subgroup analysis according to surgery type for the use of VBS versus CS in OB/GYN surgery

Outcome	Subgroup	Number of studies	Data synthesis method	Pooled outcome effect	Heterogeneity (*I*^2^)	*p* value
Operation time [min]	Total	21	Mean difference [95% CI]	– 17.08 [– 21.57, – 12.59]	87%	< 0.00001
	Hysterectomy	12		– 21.09 [– 29.42, – 12.77]	91%	< 0.00001
	Myomectomy	8		– 16.10 [– 22.16, – 10.03]	66%	< 0.00001
	Endometrioma excision	1		– 9.50 [– 15.77, – 3.23]	n/a	0.003
Suture time [min]	Total	12	Mean difference [95% CI]	– 5.39 [– 7.06, – 3.71]	93%	< 0.00001
	Hysterectomy	8		– 4.84 [– 6.88, – 2.79]	96%	< 0.00001
	Myomectomy	3		– 6.24 [– 8.29, – 4.20]	16%	< 0.00001
	Endometrioma excision	1		– 6.85 [– 8.97, – 4.73]	n/a	< 0.00001
Total post-operative complications	Total	10^a^	Risk ratio [95% CI]	1.21 [0.80, 1.84]	30%	0.36
	Hysterectomy	8^a^		1.20 [0.70, 2.05]	39%	0.50
	Myomectomy	2^a^		1.32 [0.71, 2.46]	n/a	0.37
Surgical site infection	Total (all hysterectomy)	5	Risk ratio [95% CI]	0.26 [0.09, 0.78]	0%	0.02
Estimated blood loss [ml]	Total	19	Mean difference [95% CI]	– 44.91 [– 66.01, – 23.81]	97%	0.002
	Hysterectomy	12		– 24.07 [– 39.36, – 8.79]	91%	0.001
	Myomectomy	6		– 104.95 [– 169.56, – 40.34]	95%	0.16
	Endometrioma excision	1		– 14.95 [– 35.61, 5.71]	n/a	< 0.0001
Length of stay [days]	Total	16	Mean difference [95% CI]	– 0.12 [– 0.25, 0.00]	84%	0.01
	Hysterectomy	10		– 0.24 [– 0.43, – 0.06]	82%	0.54
	Myomectomy	6		0.05 [– 0.12, 0.22]	48%	0.02
Granulation tissue	Total (all hysterectomy)	6	Risk ratio [95% CI]	0.48 [0.25, 0.89]	0%	0.02
Surgical difficulty [VAS score]	Total	4	Mean difference [95% CI]	– 1.98 [– 2.83, – 1.13]	72%	< 0.00001
	Hysterectomy	1		– 3.00 [– 3.84, – 2.16]	n/a	< 0.00001
	Myomectomy	2		– 1.91 [– 2.64, – 1.18]	18%	< 0.00001
	Endometrioma excision	1		– 1.09 [– 2.00, – 0.18]	n/a	0.02

*CI* Confidence interval

^a^One or more studies report zero events in both the study group and control group, which makes calculating the risk ratio for these studies impossible. They are therefore excluded from the calculation of pooled outcome effect

Furthermore, 8 studies compared VBS to interrupted CS, and 15 studies to running CS (Table 2). One study contained three groups and compared VBS to both running and interrupted CS [26], and only the comparison expected to show the smaller difference in primary outcomes (running CS) is included in the data synthesis. Two comparators are categorized as other CS: one study used interrupted and running sutures to close two different tissue layers [27], and one study did not further specify the type of suture [28].

**Table 2:** Outcome measures and subgroup analysis according to comparator type for the use of VBS versus CS in OB/GYN surgery

Outcome	Subgroup	Number of studies	Data synthesis method	Pooled outcome effect	Heterogeneity (I^2^)	P-value
Operation time [min]	Total	21	Mean difference [95% CI]	– 17.08 [– 21.57, – 12.59]	87%	< 0.00001
	Running suture	13		– 14.07 [– 20.99, – 7.16]	77%	< 0.0001
	Interrupted suture	6		– 25.98 [– 34.84, – 17.11]	95%	< 0.00001
	Other	2		– 13.07 [– 22.65, – 3.49]	63%	0.008
Suture time [min]	Total	12	Mean difference [95% CI]	– 5.39 [– 7.06, – 3.71]	93%	< 0.00001
	Running suture	10		– 5.32 [– 7.42, – 3.22]	94%	< 0.00001
	Interrupted suture	2		– 5.89 [– 8.83, – 2.95]	94%	< 0.0001
Total post-operative complications	Total	10^a^	Risk ratio [95% CI]	1.21 [0.80, 1.84]	30%	0.36
	Running suture	7^a^		1.11 [0.76, 1.61]	0%	0.60
	Interrupted suture	3		1.44 [0.42, 4.93]	74%	0.56
Surgical site infection	Total	5	Risk ratio [95% CI]	0.26 [0.09, 0.78]	0%	0.02
	Running suture	3		0.36 [0.07, 1.91]	0%	0.23
	Interrupted suture	2		0.21 [0.05, 0.88]	0%	0.03
Estimated blood loss [ml]	Total	19	Mean difference [95% CI]	– 44.91 [– 66.01, – 23.81]	97%	< 0.0001
	Running suture	12		– 32.23 [– 53.00, – 11.46]	91%	0.002
	Interrupted suture	5		– 73.12 [– 127.58, – 18.66]	98%	0.009
	Other	2		– 45.79 [– 157.40, 65.83]	59%	0.42
Length of stay [days]	Total	16	Mean difference [95% CI]	– 0.12 [– 0.25, 0.00]	84%	0.06
	Running suture	8		– 0.03 [– 0.21, 0.16]	53%	0.76
	Interrupted suture	6		– 0.15 [– 0.34, 0.04]	92%	0.12
	Other	2		– 0.84 [– 2.21, 0.53]	88%	0.23
Granulation tissue	Total	6	Risk ratio [95% CI]	0.48 [0.25, 0.89]	0%	0.02
	Running suture	1		2.39 [0.13, 44.09]	n/a	0.56
	Interrupted suture	4		0.44 [0.19, 0.98]	0%	0.05
	Other	1		0.45 [0.16, 1.28]	n/a	0.13
Surgical difficulty [VAS score]	Total (all running suture)	4	Mean difference [95% CI]	– 1.98 [– 2.83, – 1.13]	72%	< 0.00001

*CI* Confidence interval

^a^One or more studies report zero events in both the study group and control group, which makes calculating the risk ratio for these studies impossible. They are therefore excluded from the calculation of pooled outcome effect

### Risk of bias of included studies

Risk of bias was assessed using the Downs and Black checklist for quality assessment, which can be applied to RCTs and non-RCTs. Overall, a medium risk of bias was found, with 4 studies rated “good”, 15 studies rated “fair”, and 6 studies rated “poor”. No studies were rated “excellent” (Table S2).

Several factors likely influenced the risk of bias. One factor is the intrinsic inability to blind surgeons to the device used for wound closure, and only one study blinded the person who assesses the outcomes [5]. More than half of the included studies (*n* = 13) were retrospective, and more than half of the prospective studies (*n* = 6) were not randomized. One study recorded the outcomes for the study group prospectively, but used a historic comparator population, leaving only five RCTs among the included studies. Finally, many studies did not perform power calculations and/or were underpowered to detect outcomes other than differences in operation and/or suture time.

### Synthesis of results

All outcomes are summarized in Tables 1 and 2.

#### Operation time

Operation time was reported by 22 studies. López et al. [29] only reported a difference of 6 min (95% CI − 2.02; 15.4, *p* = not significant) between the VBS group and the CS group without stating the actual operation time, and was therefore not included in the data synthesis.

**Fig. 2:**
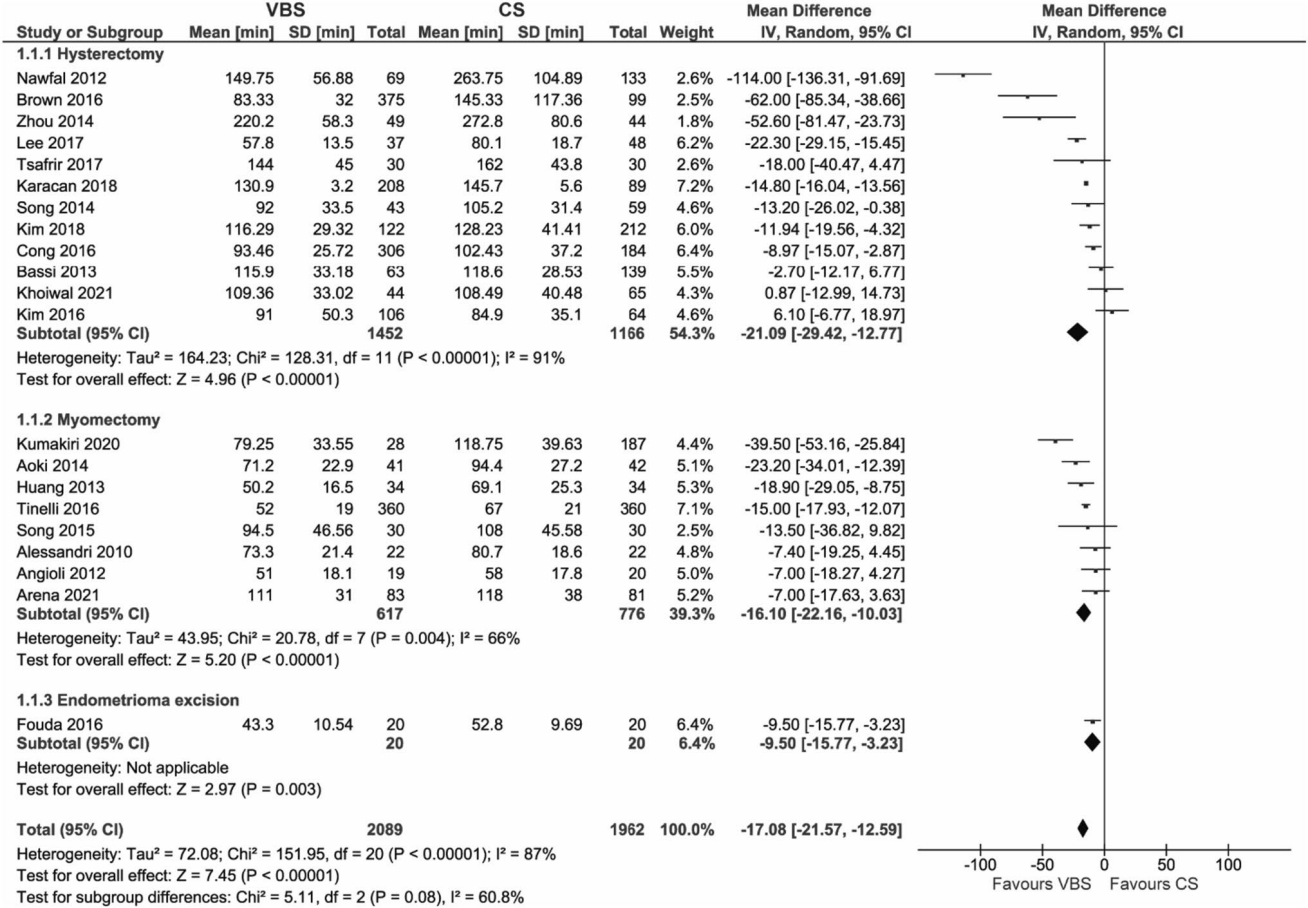
Pooled estimates of operation time [min] and subgroup analysis according to surgery type for the use of VBS versus CS in OB/GYN surgery. Data extracted from references: 8, 9, 26–28, 31, 32, 39–51

The use of VBS instead of CS reduced operation time by 17.08 min (95% CI – 21.57; – 12.59, *p* < 0.00001). The reduction in operation time was observed for all surgery types (Fig. 2) and all comparator types, where it was most pronounced in comparison to interrupted sutures (Figure S1).

This outcome showed substantial heterogeneity (*I*^2^ = 87%).

#### Suture time

**Fig. 3:**
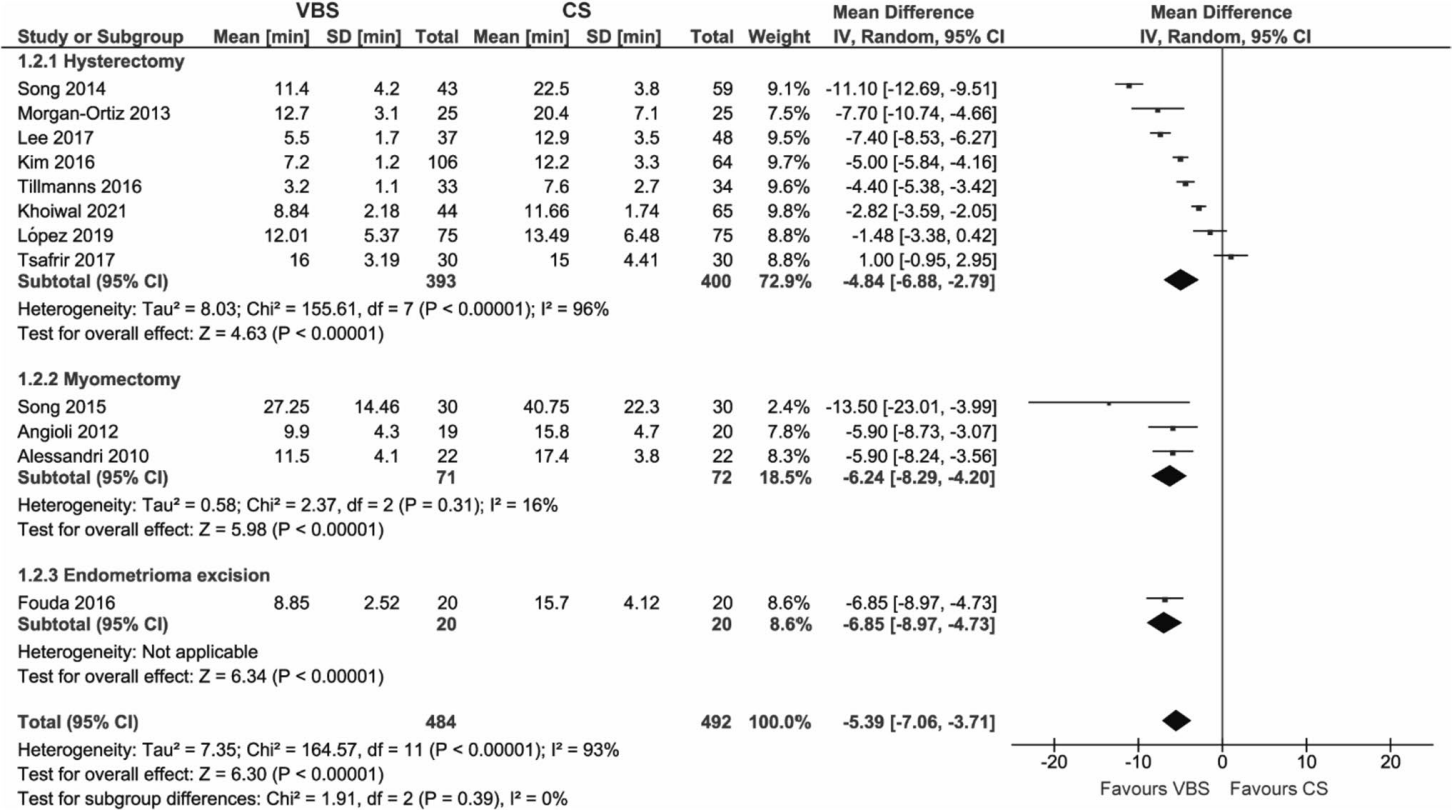
Pooled estimates of suture time [min] and subgroup analysis according to surgery type for the use of VBS versus CS in OB/GYN surgery. Data extracted from references: 8, 9, 26, 29–31, 37, 40, 44, 47, 49

Suture time was reported in 12 studies. Using VBS instead of CS reduced suture time by 5.39 min (95% CI – 7.06; – 3.71, *p* < 0.00001). This reduction was observed for all analyzed subgroups (Fig. 3 and Figure S2).

This outcome showed substantial heterogeneity (*I*^2^ = 93%).

#### Total post-operative complications

The number of total post-operative complications was extracted from 10 studies. Two studies reported that no post-operative complications were observed in either group and therefore excluded from the data synthesis [30, 31].

**Fig. 4:**
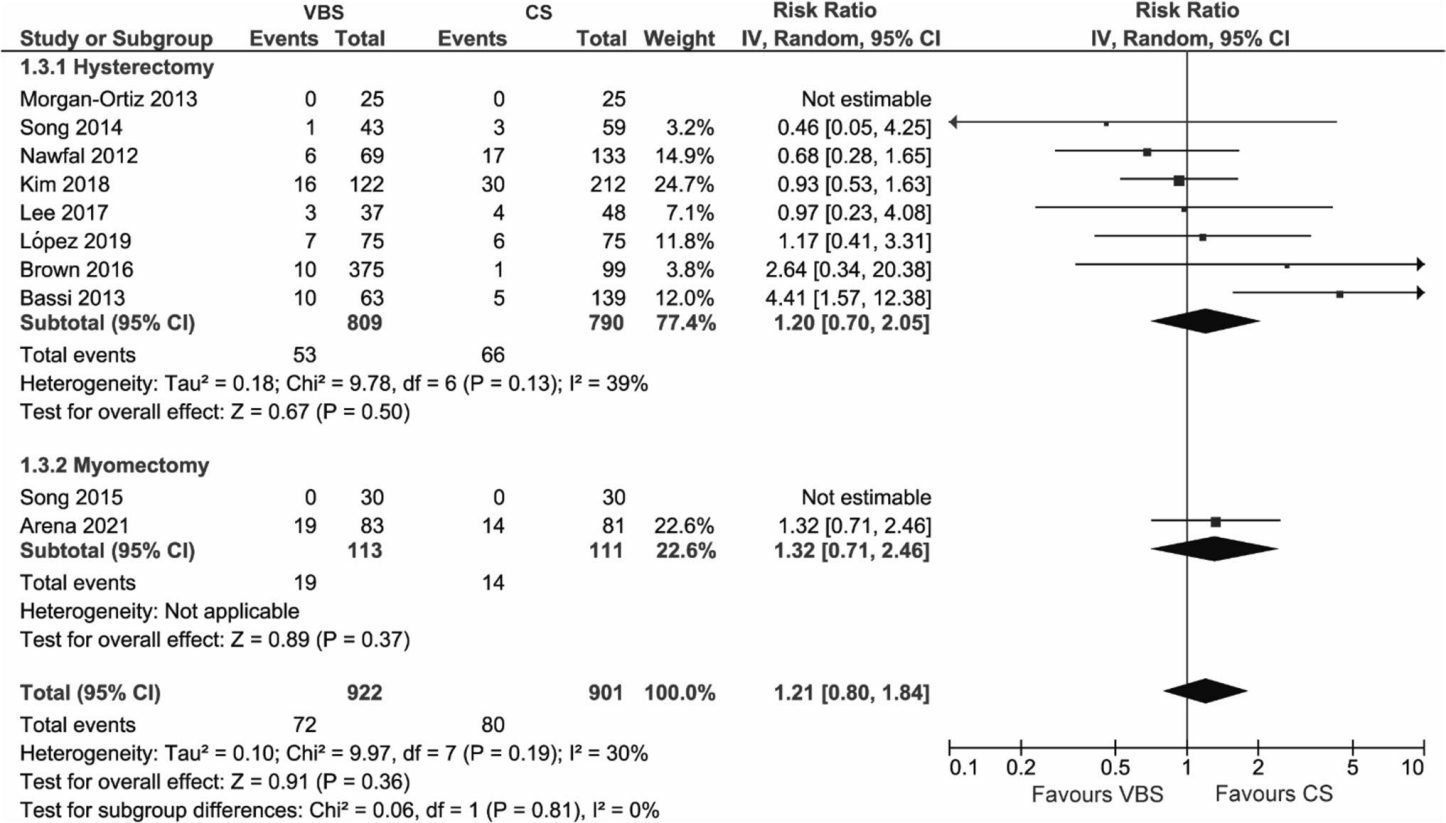
Pooled estimates of total post-operative complications and subgroup analysis according to surgery type for the use of VBS versus CS in OB/GYN surgery. Data extracted from references: 29–32, 42, 43, 45, 47–49

The rate of total post-operative complications showed no significant difference between VBS and CS (RR 1.21, 95% CI 0.80; 1.84, *p* = 0.36). This held true in all analyzed subgroups (Fig. 4 and Figure S3).

The outcome did not show substantial heterogeneity (*I*^2^ = 30%).

#### Surgical site infections

A total of five studies reported surgical site infections, all of them on hysterectomy.

**Fig. 5:**
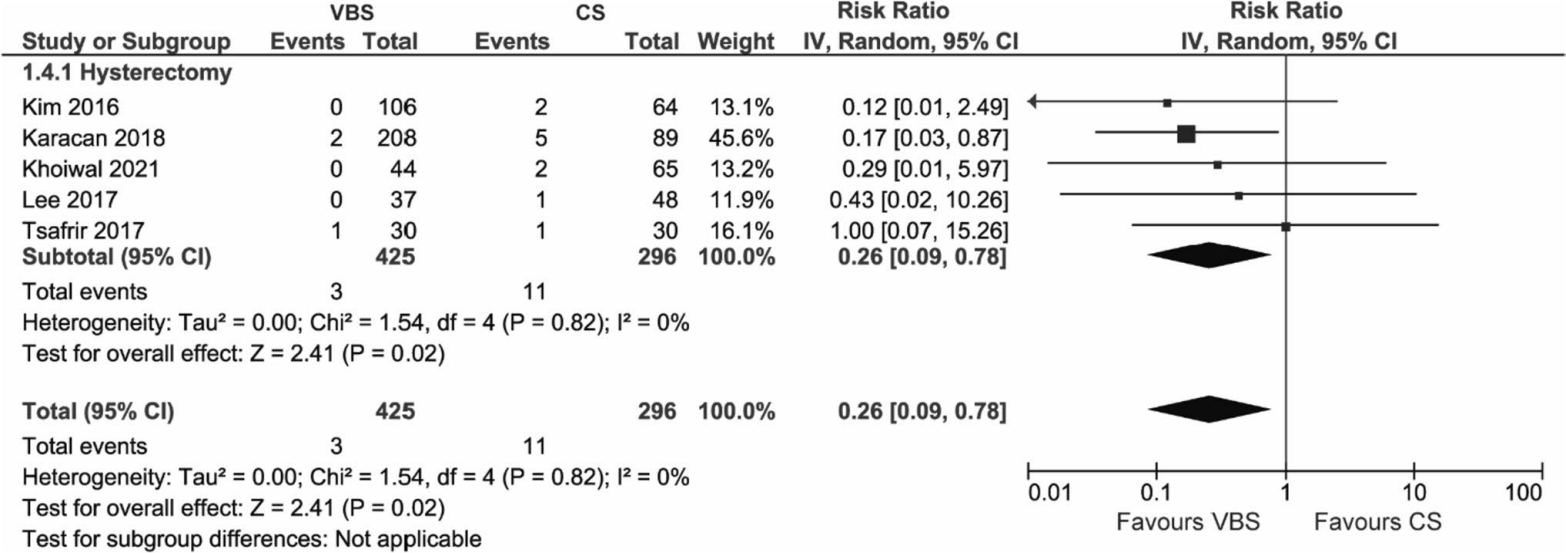
Pooled estimates of surgical site infections and subgroup analysis according to surgery type for the use of VBS versus CS in OB/GYN surgery. Data extracted from references: 8, 26, 39, 44, 47

VBS were found to significantly reduce surgical site infections compared to CS (RR 0.26, 95% CI 0.09; 0.78, *p* = 0.02) (Fig. 5). The subgroup analysis by comparator type showed that VBS reduced surgical site infections in comparison to interrupted sutures (RR 0.21, 95% CI 0.05; 0.88, *p* = 0.03) but not running sutures (RR 0.36, 95% CI 0.07; 1.91, *p* = 0.23) (Figure S4).

The outcome did not show any heterogeneity (*I*^2^ = 0%).

#### Estimated blood loss

The estimated blood loss was reported in 19 studies.

**Fig. 6:**
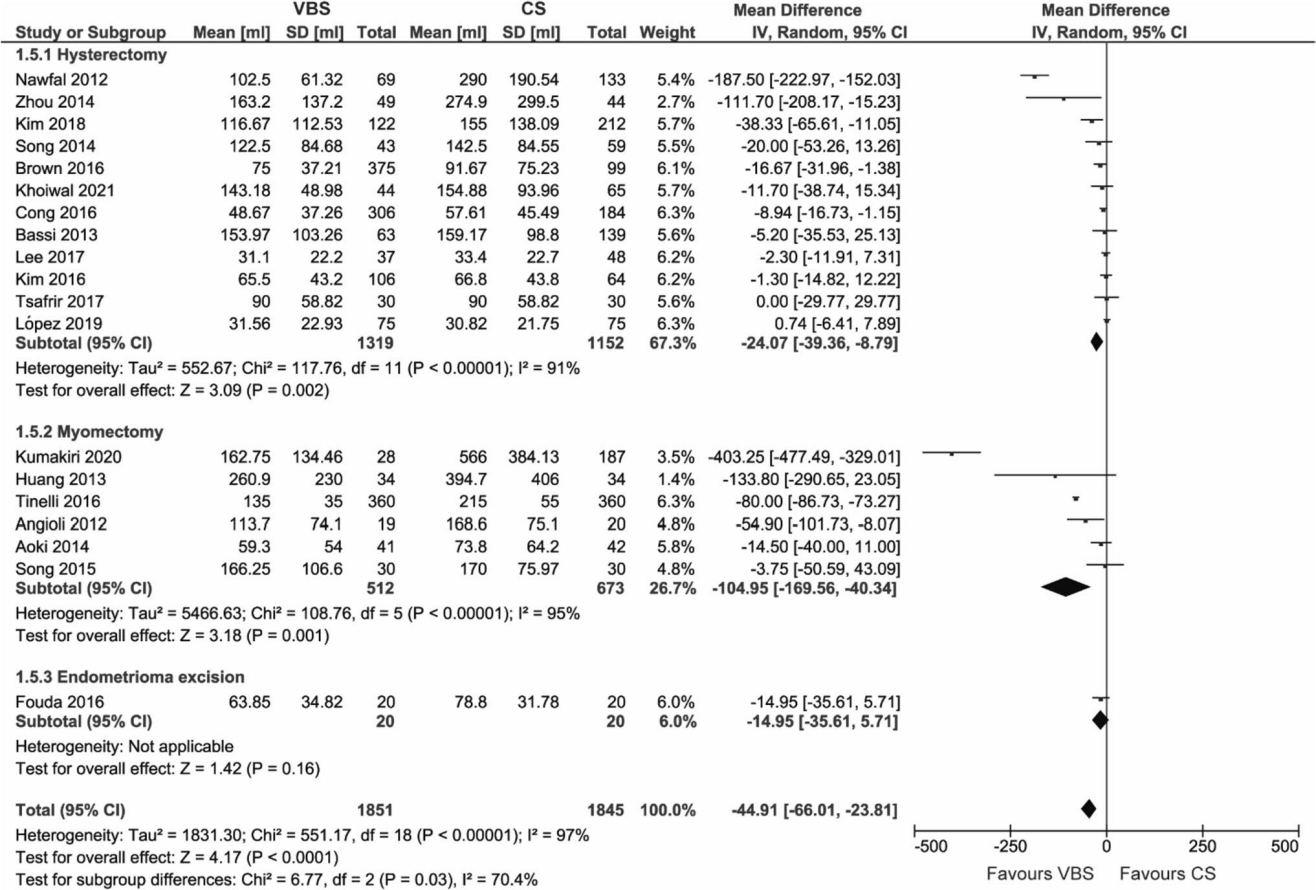
Pooled estimates of estimated blood loss [ml] and subgroup analysis according to surgery type for the use of VBS versus CS in OB/GYN surgery. Data extracted from references: 8, 26–29, 31, 32, 40, 41, 43–51

Overall, the estimated blood loss was reduced by 44.91 ml (95% CI – 66.01; – 23.81, *p* < 0.0001) with VBS compared to CS (Fig. 6) and showed significant differences between the procedure subgroups. While no significant difference was found in excision of endometrioma (WMD – 14.95 ml, 95% CI – 35.61; 5.71, *p* = 0.16), VBS significantly reduced the blood loss during hysterectomy (WMD – 24.07 ml, 95% CI – 39.36; – 8.79, *p* = 0.002) and myomectomy (WMD – 104.95 ml, 95% CI – 169.56; – 40.34, *p* = 0.001).

In the subgroup analysis by comparator, blood loss was reduced in comparison to running and interrupted CS, but not compared to the subgroup defined as other CS (Figure S5).

This outcome showed substantial heterogeneity (*I*^2^ = 97%).

#### Length of hospital stay

A total of 17 studies reported the length of hospital stay.

**Fig. 7:**
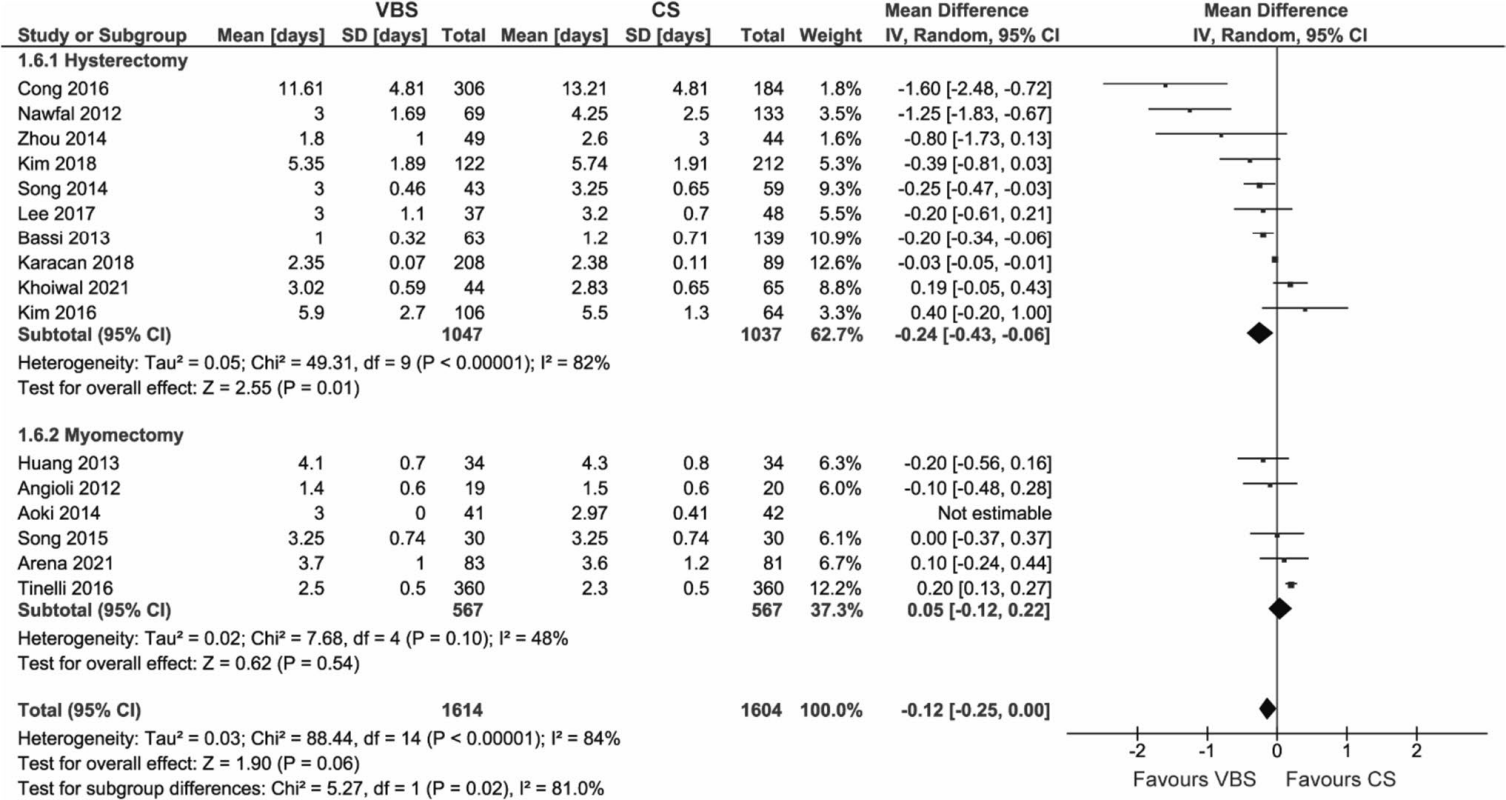
Pooled estimates of length of hospital stay [days] and subgroup analysis according to surgery type for the use of VBS versus CS in OB/GYN surgery. Data extracted from references: 8, 27, 28, 31, 40–45, 47–51

Overall, the length of stay was not significantly different between VBS and CS (WMD – 0.12 days, 95% CI – 0.25; 0.00, *p* = 0.06) (Fig. 7). VBS led to a reduced length of hospital stay in hysterectomy (WMD – 0.24 days, 95% CI – 0.43; – 0.06, *p* = 0.01); all other subgroups show no significant effect (Figure S6).

The outcome showed substantial heterogeneity (*I*^2^ = 84%).

Brown et al. [32] reported a significant difference in the percentage of patients who left the hospital on a certain day instead of the actual length of stay and so were not included in the data synthesis. While a larger percentage of patients in the VBS group (23.1%) left the hospital on the same day as the procedure than in the CS group (14.1%), all patients who stayed for 3 or more days were in the study group (3.8%). This makes overall interpretation difficult.

#### Formation of granulation tissue

The formation of granulation tissue is reported in six studies on hysterectomy.

**Fig. 8:**
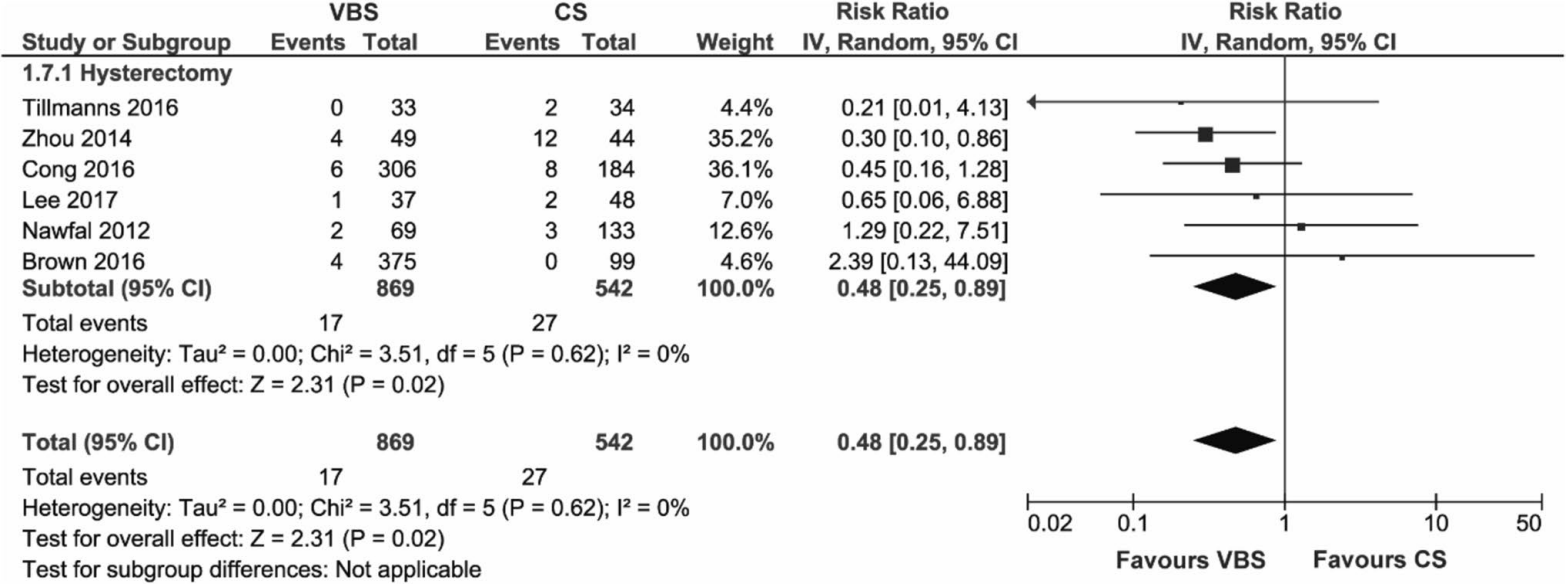
Pooled estimates of formation of granulation tissue and subgroup analysis according to surgery type for the use of VBS versus CS in OB/GYN surgery. Data extracted from references: 28, 32, 37, 47, 48, 51

The use of VBS significantly reduced the formation of granulation tissue compared to CS (RR 0.48, 95% CI 0.25; 0.89, *p* = 0.02) (Fig. 8). The subgroup analysis showed a significant reduction compared to interrupted CS (RR 0.44, 95% CI 0.19; 0.98, *p* = 0.05), but not compared to running CS (RR 2.39, 95% CI 0.13; 44.09, *p* = 0.56) or the subgroup defined as other CS (RR 0.45, 95% CI 0.16; 1.28, *p* = 0.13) (Figure S7).

The outcome did not show any heterogeneity (*I*^2^ = 0%).

#### Surgical difficulty

The surgical difficulty was assessed in four studies, all comparing VBS to running CS.

**Fig. 9:**
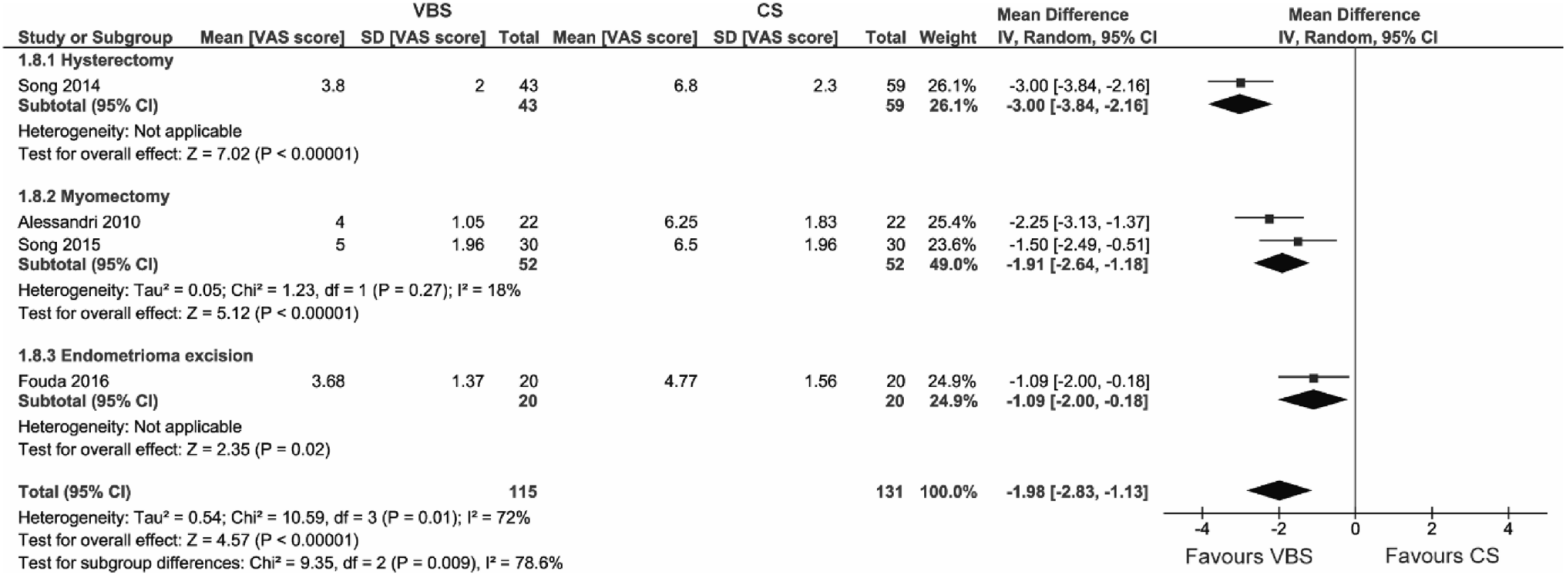
Pooled estimates of surgical difficulty [VAS score] and subgroup analysis according to surgery type for the use of VBS versus CS in OB/GYN surgery. Data extracted from references: 5, 9, 31, 49

VBS significantly reduced the surgical difficulty in comparison to running CS (WMD – 1.98 VAS score points, 95% CI – 2.83; – 1.13, *p* < 0.00001). This effect was observed across all types of surgery in the subgroup analysis (Fig. 9).

The outcome showed substantial heterogeneity (*I*^2^ = 72%).

### Risk of bias due to missing data

Potential biases such as publication or reporting bias were assessed using funnel plot analysis (Figure S9). The distribution of studies reporting suture time, total post-operative complications, surgical site infections, granulation tissue formation, and surgical difficulty was overall symmetrical, indicating little risk of bias.

The funnel plots for operation time, estimated blood loss, and length of hospital stay are less symmetrical, possibly due to heterogeneity, which is displayed to a high degree by these outcomes. The different surgical procedures and approaches (laparoscopic, robot-assisted, or mini-laparotomy) are likely large contributors to heterogeneity.

It could also indicate a publication bias affecting these outcomes. As suggested by Sterne et al. [33], we repeated the analysis of outcomes with asymmetric distribution and evidence of between-study heterogeneity using the fixed instead of the random effects model. While the overall effect sizes changed slightly, neither the direction nor the significance of the pooled effects changed (data not shown).

### Sensitivity analyses

**Fig. 10:**
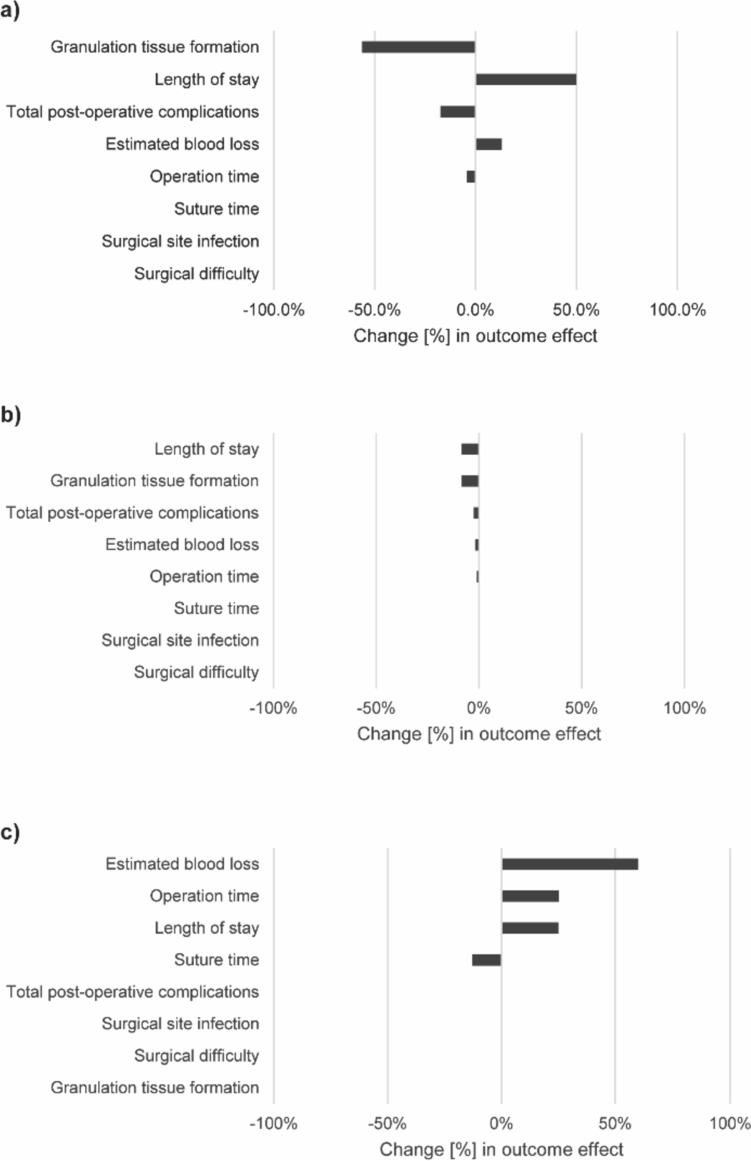
Effect of sensitivity analyses on pooled estimates of outcomes. Presented are the changes in pooled outcome estimates in percent if **a**) all studies rated “poor” were excluded from the analysis, **b**) the top 10% (*n* = 3) studies with the largest patient population were excluded from the analysis, and **c**) studies reporting outlier values for each outcome were excluded from the analysis

The results of the sensitivity analyses are presented in Fig. 10 and Table 3.

**Table 3:** Comparison of pooled effect between original complete meta-analysis and all sensitivity analyses

Outcome	Data synthesis method	Pooled effect of complete meta-analysis	Pooled effect of sensitivity analyses			Change to pooled effect			Difference in effect direction
			Study quality	Study size	Outliers	Study quality (%)	Study size (%)	Outliers (%)	
Operation time [min]	WMD [95% CI]	– 17.08 [– 21.57, – 12.59]	– 16.34 [– 21.29, – 11.38]	– 17.25 [– 23.05, – 11.45]	– 12.77 [– 15.30, – 10.25]	– 4.3	– 1.0	25.2	No
Suture time [min]	WMD [95% CI]	– 5.39 [– 7.06, – 3.71]	– 5.39 [– 7.06, – 3.71]	– 5.39 [– 7.06, – 3.71]	– 6.08 [– 7.20, – 4.96]	0.0	0.0	– 12.8	No
Total post-operative complications	RR [95% CI]	1.21 [0.80, 1.84]	1.00 [0.71, 1.40]	1.18 [0.76, 1.83]	1.21 [0.80, 1.84]	– 17.4	– 2.5	0.0	No
Surgical site infection	RR [95% CI]	0.26 [0.09, 0.78]	0.26 [0.09, 0.78]	0.23 [0.09, 0.78]	0.26 [0.09, 0.78]	0.0	0.0	0.0	No
Estimated blood loss [ml]	WMD [95% CI]	– 44.91 [– 66.01, – 23.81]	– 50.75 [– 78.05, – 23.45]	– 45.76 [– 67.64, – 23.87]	– 17.94 [– 27.33, – 8.55]	13.0	– 1.9	60.1	No
Length of stay [days]	WMD [95% CI]	– 0.12 [– 0.25, 0.00]	– 0.06 [– 0.20, 0.08]	– 0.13 [– 0.26, – 0.01]	– 0.09 [– 0.19, 0.01]	50.0	– 8.3	25.0	Yes (study size)
Granulation tissue formation	RR [95% CI]	0.48 [0.25, 0.89]	0.75 [0.21, 2.71]	0.44 [0.19, 0.98]	0.48 [0.25, 0.89]	– 56.3	– 8.3	0.0	Yes (study quality)
Surgical difficulty [VAS score]	WMD [95% CI]	– 1.98 [– 2.83, – 1.13]	– 1.98 [– 2.83, – 1.13]	– 1.98 [– 2.83, – 1.13]	– 1.98 [– 2.83, – 1.13]	0.0	0.0	0.0	No

*CI* Confidence interval, *RR* Risk ratio, *WMD* Weighted mean difference

Studies with a high risk of bias mainly affected the pooled effect for granulation tissue formation, which no longer showed a significant difference in the sensitivity analysis (Fig. 10a, Table 3). Furthermore, low-quality studies influenced the length of hospital stay, total post-operative complications, estimated blood loss, and operation time but without changing the direction of the pooled effect.

The sensitivity analysis for study size excluded the 10% (*n* = 3) largest studies, which account for a total of 1684 patients or 37.6% of the pooled population. Study size had a small effect on length of stay and granulation tissue formation, and a negligible effect on total post-operative complications, estimated blood loss, and operation time (Fig. 10b, Table 3). Only the pooled effect for length of stay changed direction in the sensitivity analysis: it showed a barely significant reduction that was not present in the full meta-analysis.

Studies reporting outlier results had a large effect on estimated blood loss, and a smaller effect on operation time, length of stay, and suture time (Fig. 10c, Table 3). However, the direction of the pooled effect was not affected in any of these outcomes.

Overall, the sensitivity analyses showed that the results were mostly robust and consistent. The only result that should be interpreted with caution is the reduction of granulation tissue formation in the VBS group, as it was mainly due to results reported in low-quality studies.

## Discussion

This meta-analysis included 25 comparative studies comprising 4452 women undergoing gynecological surgery whose incisions were closed with either VBS or CS. The analysis showed that the use of VBS resulted in significantly shorter operation time and suture time, significantly reduced blood loss, surgical site infection, and surgical difficulty. A significant reduction in granulation tissue formation was also observed; however, this result is based on low-quality studies and should therefore be interpreted with caution.

Other outcomes of interest showed no difference between the groups. Importantly, no outcomes were identified where VBS performed worse than CS.

Barbed sutures were designed to eliminate knot-tying, one of the most time-consuming and challenging tasks in laparoscopic surgery [4, 5]. The present meta-analysis confirmed that VBS reduce suture time and operation time as well as surgical difficulty. Other meta-analyses based on pooled data of different barbed suture types in OB/GYN surgery have reported similar results regarding the efficacy and safety of barbed sutures [14, 15, 18, 34, 35]. The approx. 5 min reduction in suture time reported here is unlikely to be clinically significant, but can be considered an indicator for a simplified surgery and may translate into a risk reduction. In contrast, a reduction of the operative time by approx. 17 min can result in large time savings and may well have clinical significance.

The reduction in operation time associated with VBS is more pronounced in comparison to interrupted than running CS, as running CS require fewer knots and are therefore faster to do. Interestingly, this effect is much less pronounced for suture time, where the time savings VBS provide are very similar for both interrupted and running CS. This may be due to the small number of studies with interrupted CS (*n* = 2) that reported suture time.

The reduced suturing time may explain the reduction in estimated blood loss that was observed with VBS: faster wound closure stops bleeding earlier, thus reducing blood loss.

Barbed sutures are not only fast and efficient, but also help to avoid the complications that surgical knots can cause [6]. In the present meta-analysis, VBS were associated with a reduction in surgical site infection and formation of granulation tissue only in comparison to interrupted CS which have frequent knots, not compared to running CS only secured at both ends. Surgical knots reduce the tensile strength of sutures, are a focal point for tissue stress, and introduce the highest density of foreign body material in a suture line, which can cause an inflammatory reaction [4, 6, 7]. In contrast, knotless barbed sutures maintain tension and equally distribute the tensile strength along the suture line, thereby reducing stress and potentially improving wound healing [4, 6].

VBS generally have higher material costs than CS [9, 26, 36], but the reduction in operative time they provide translates into substantial savings in operating room costs [37]. A formal cost-effectiveness analysis is needed to answer the question of economics. To the best of our knowledge, no such economic analysis of barbed sutures in OB/GYN surgery is currently available.

Previously, cases of small bowel obstructions have been reported after vaginal cuff closure and myomectomy with barbed sutures, among other procedures [38]. No bowel complications were observed in any of the studies included in the present meta-analysis, possibly because it might be a rare complication [36] and can likely be avoided by cutting the free end of the barbed suture flush with the tissue so that the barbs on the remnant suture material cannot entrap neighboring tissue [8, 38, 39].

## Strengths and limitations

Compared to previous meta-analyses regarding barbed sutures in OB/GYN surgeries, this study takes both a broader and a narrower approach. On one hand, it provides a broader overview, as it includes non-RCT comparative studies in addition to RCTs as well as several different surgical procedures and comparators. The subgroup analyses ensured that no nuances were lost by pooling the results across different surgeries and comparators. On the other hand, our meta-analysis has a narrower focus, as it is (to our knowledge) the first meta-analysis to focus on a specific type of barbed suture by design.

This study has several limitations. Firstly, although the inclusion of non-RCTs provided additional data that can still be clinically meaningful, it also led to an increased risk of bias. The majority of studies included in this meta-analysis were only rated “fair” according to the Downs and Black checklist for quality assessment, indicating an overall risk of bias that should be kept in mind when interpreting the results. Further bias was potentially introduced by studies that did not report results as mean and standard deviation, as we had to estimate them from other variables (such as median and range) in order to include them in the analysis. Secondly, while this meta-analysis included relatively commonly reported complications such as surgical site infection or formation of granulation tissue, no analysis of rarer complications such as wound dehiscence was possible (e.g., wound dehiscence was not reported for myomectomy in any of the included studies). Most included studies were underpowered to detect them and/or make a reliable distinction between the occurrence in the study and comparator groups, and they may be at a high risk of reporting bias. Larger studies are needed to better understand the interplay between the use of barbed sutures and rare complications. Thirdly, although the inclusion of different study designs, surgical procedures, and comparators provided a broad overview of the field of gynecologic surgery in its entirety, it also introduced a high degree of heterogeneity. Further heterogeneity was potentially introduced by the lack of standards for outcome reporting. Commonly reported outcomes are often not defined in the studies, making it unclear if the data derived from different studies describes the same measure. A random effects model was used to reduce the ensuing effects of heterogeneity but cannot eliminate them. Finally, although obstetric procedures were explicitly part of this study, the search did not identify any relevant publications. Only two databases were searched in this systematic review, and unpublished studies were also not considered, potentially missing relevant results.

## Conclusions

In conclusion, this meta-analysis shows that V-Loc^™^ barbed sutures are safe and effective in gynecological surgeries. They are associated with many benefits, including reductions in operation and suture time, blood loss, and surgical difficulty. The use of barbed sutures facilitates suturing in laparoscopic myomectomy, hysterectomy, and excision of endometrioma. A health-economic analysis is required to settle the question if the increased material costs of barbed sutures are covered by the savings associated with the reductions in operation time, blood loss, and surgical site infection in gynecological procedures.

### Supplementary Information

Below is the link to the electronic supplementary material.

Supplementary file1 (DOCX 3749 KB)

Supplementary file2 (PDF 246 KB)

Supplementary file3 (DOCX 34 KB)

## Data Availability

The template data collection form, the data extracted from included studies, and the data used for all analyses are available from the authors upon reasonable request.
