# Supplementary material for: Efficacy and safety of V-Loc™ barbed sutures versus conventional suture techniques in gynecological surgery: a systematic review and meta-analysis
Source: Arch Gynecol Obstet. 2023 Dec 21;309(4):1249–65. doi: 10.1007/s00404-023-07291-3 (PMC10894094; doi:10.1007/s00404-023-07291-3)
Supplement: Supplementary file 1 — Supplementary file1 (DOCX 3749 KB) [file 404_2023_7291_MOESM1_ESM.docx]

## Supplementary material to

**Efficacy and Safety of V-Loc™ Barbed Sutures Versus Conventional Suture Techniques in Gynecological Surgery: A Systematic Review and Meta-Analysis.**

## Journal

Archives of Gynecology and Obstetrics

## Authors

Juliane Hafermann (1)

Ubong Silas (1)

Rhodri Saunders (1)

## Author affiliations

1. Coreva Scientific GmbH & Co. KG, Koenigswinter, Germany

## Corresponding author

Juliane Hafermann

ORCiD ID: 0009-0004-4595-7794

Coreva Scientific GmbH & Co KG, Im Muehlenbruch 1, 53639 Koenigswinter, Germany

Tel +49 2223 781 8010

Fax +49 761 76 999 421

Email [ourpublications@coreva-scientific.com](mailto:ourpublications@coreva-scientific.com)

## Supplementary figures

#### **Fig. S1**

Pooled estimates of operation time [min] and subgroup analysis according to comparator type for the use of VBS versus CS in OB/GYN surgery


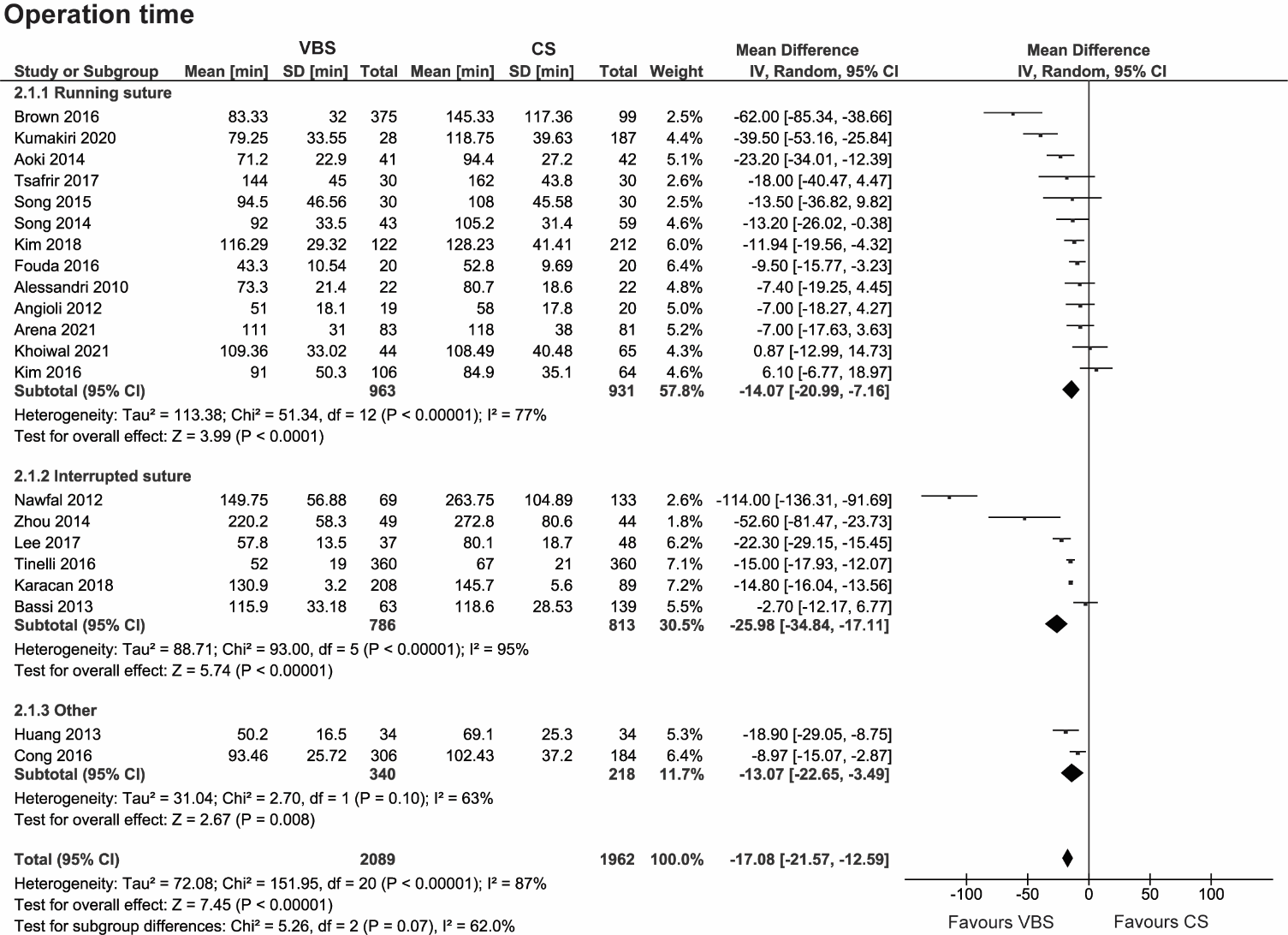


#### **Fig. S2**

Pooled estimates of suture time [min] and subgroup analysis according to comparator type for the use of VBS versus CS in OB/GYN surgery


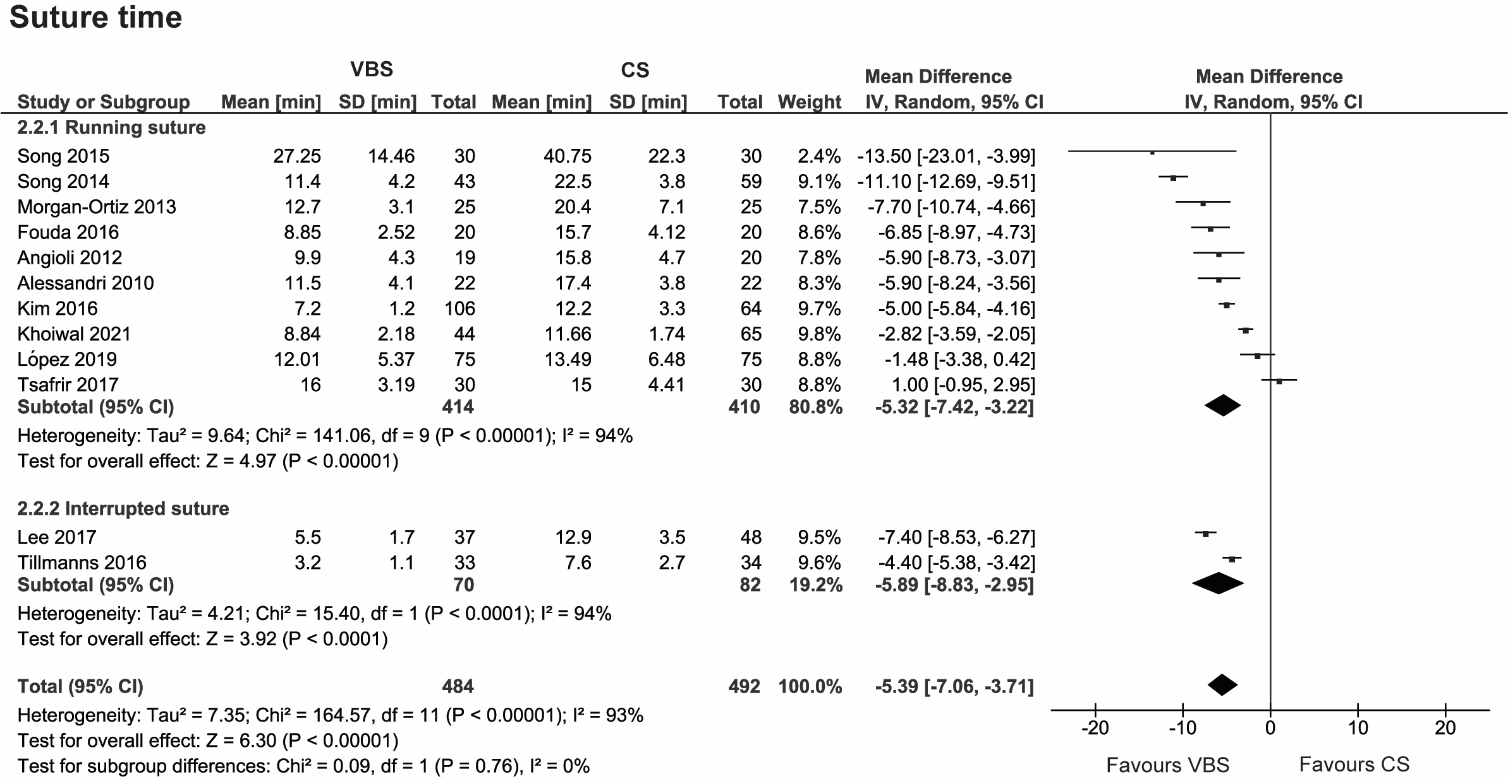


#### **Fig. S3**

Pooled estimates of total post-operative complications and subgroup analysis according to comparator type for the use of VBS versus CS in OB/GYN surgery


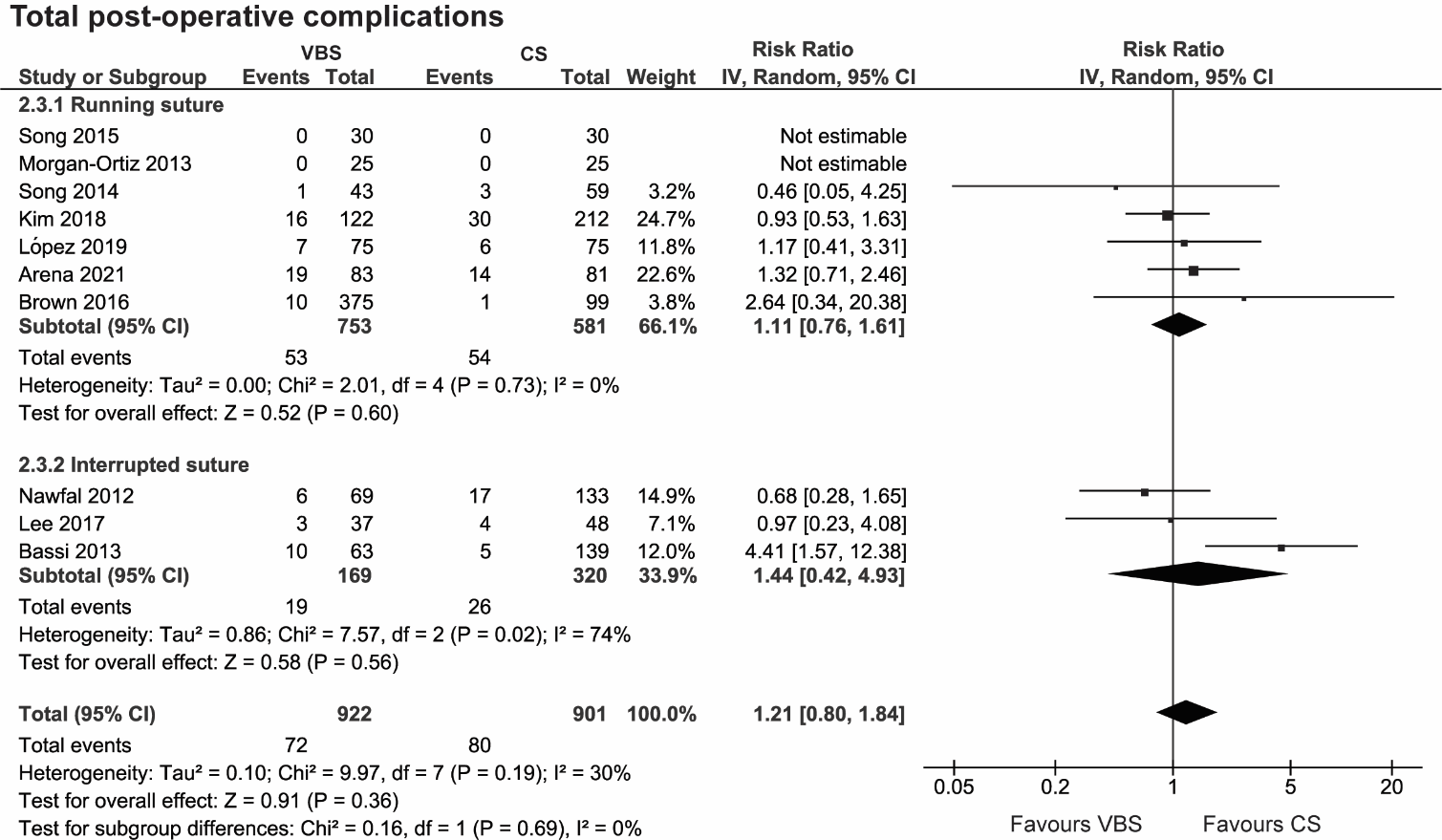


#### **Fig. S4**

Pooled estimates of surgical site infections and subgroup analysis according to comparator type for the use of VBS versus CS in OB/GYN surgery


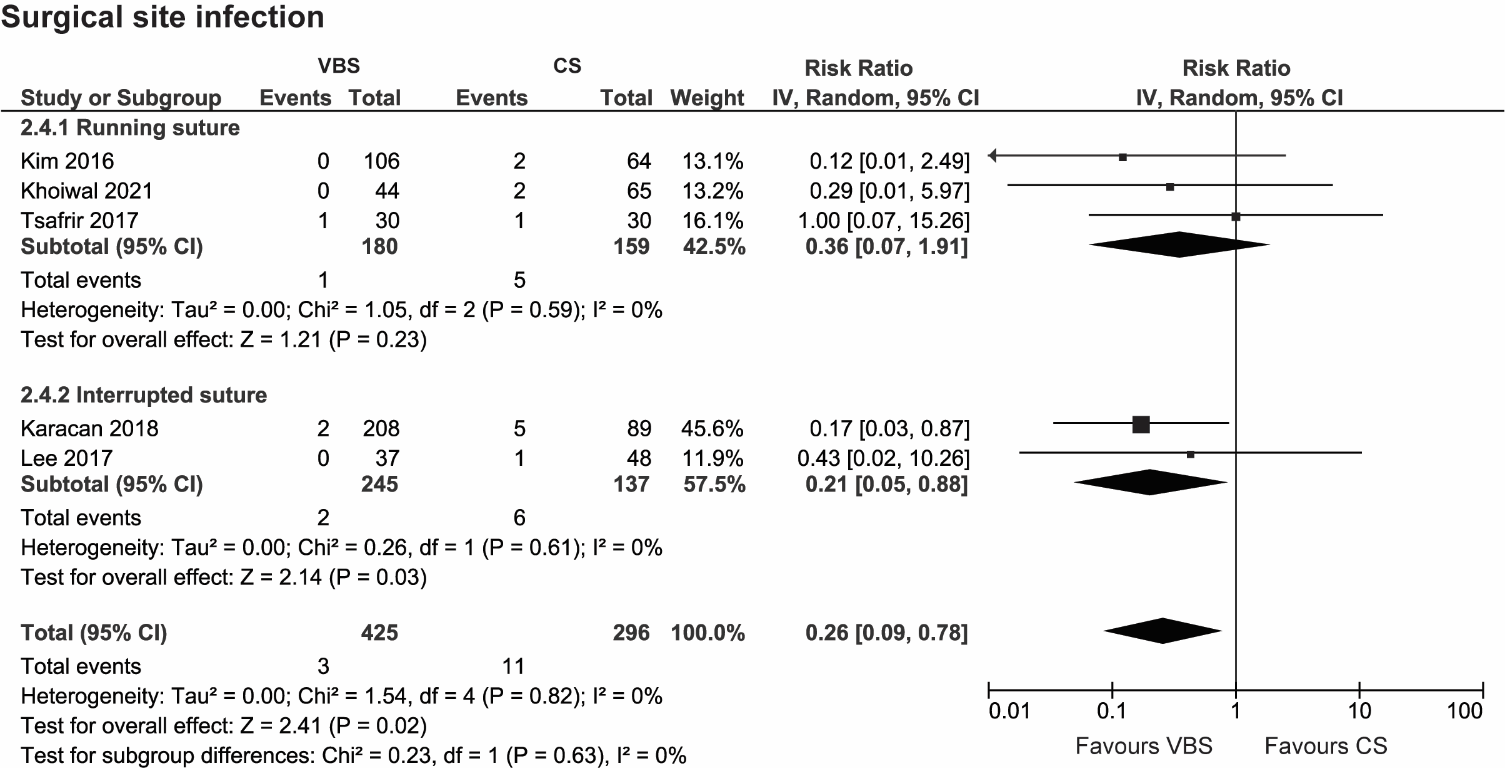


#### **Fig. S5**

Pooled estimates of estimated blood loss [ml] and subgroup analysis according to comparator type for the use of VBS versus CS in OB/GYN surgery


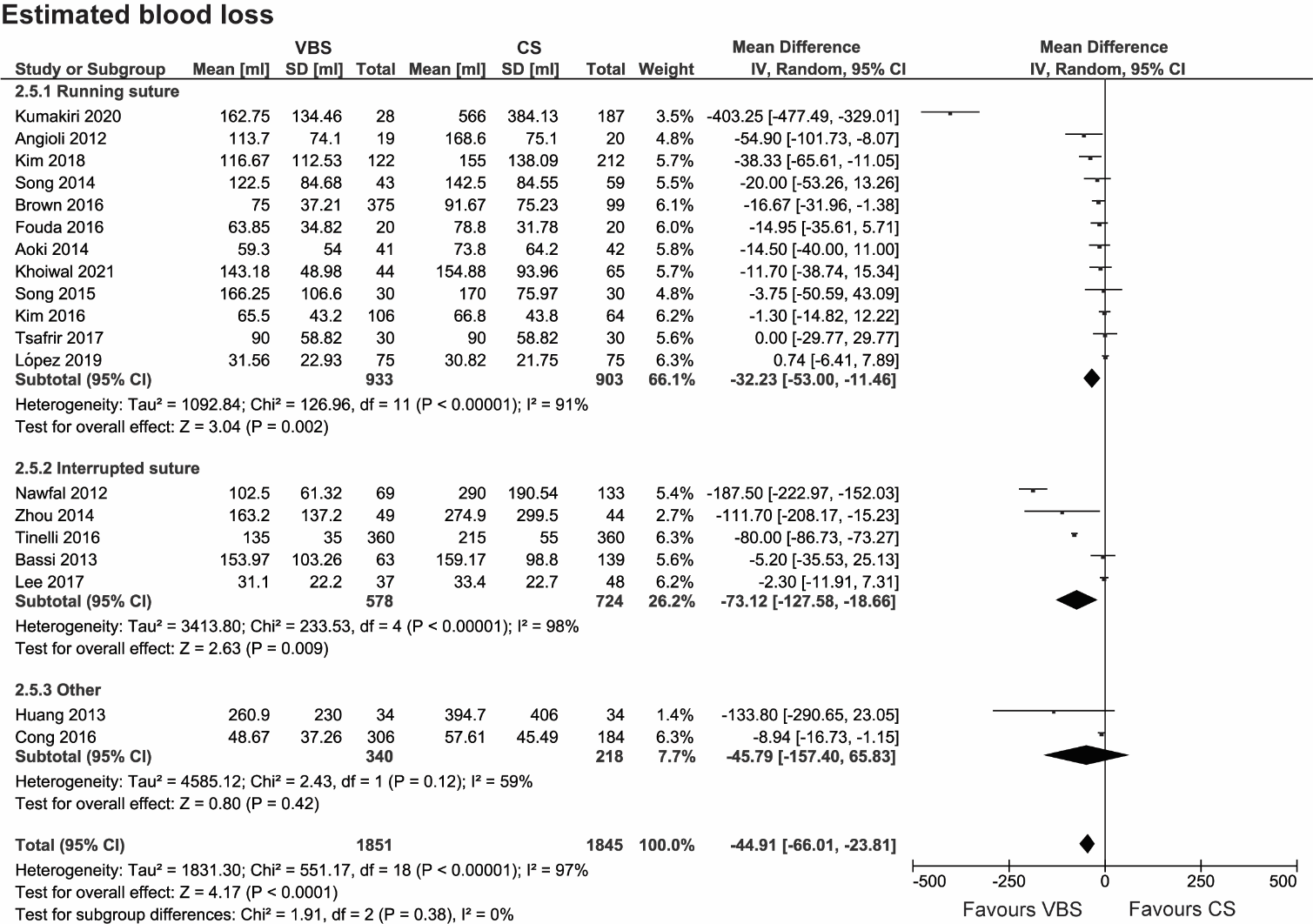


#### **Fig. S6**

Pooled estimates of length of hospital stay [days] and subgroup analysis according to comparator type for the use of VBS versus CS in OB/GYN surgery


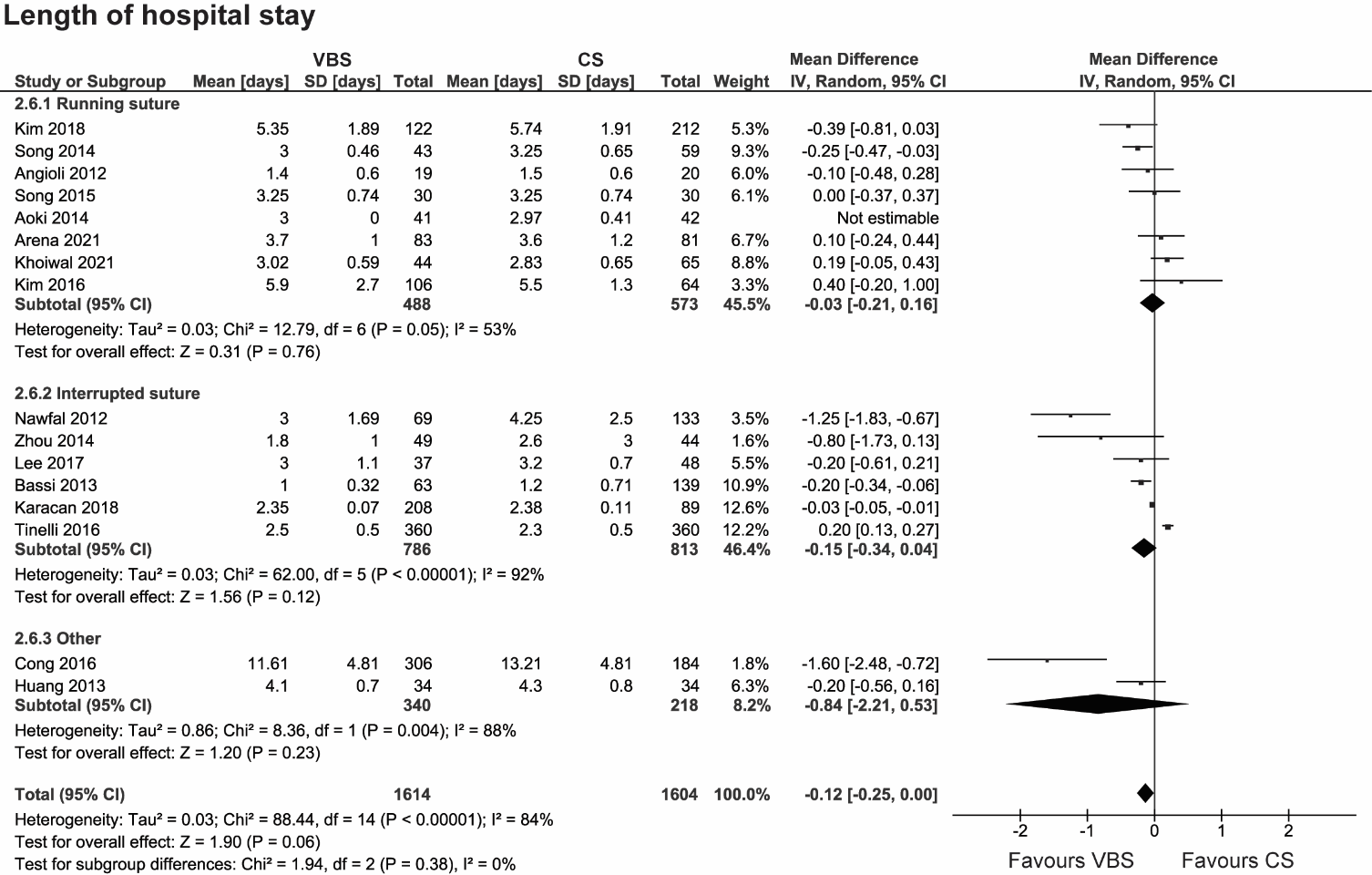


#### **Fig. S7**

Pooled estimates of formation of granulation tissue and subgroup analysis according to comparator type for the use of VBS versus CS in OB/GYN surgery


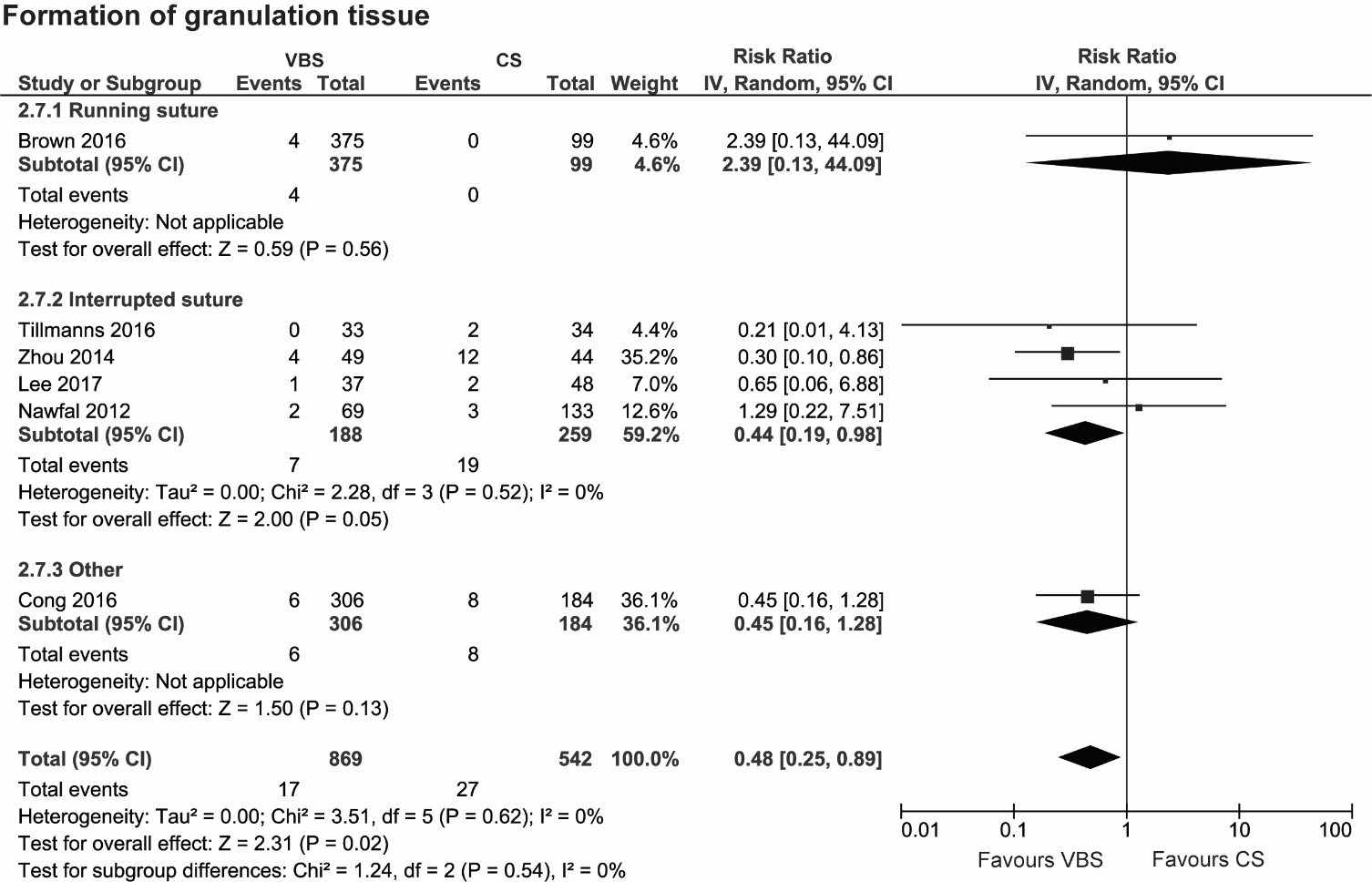


#### **Fig. S8**

Pooled estimates of surgical difficulty [VAS score] and subgroup analysis according to comparator type for the use of VBS versus CS in OB/GYN surgery


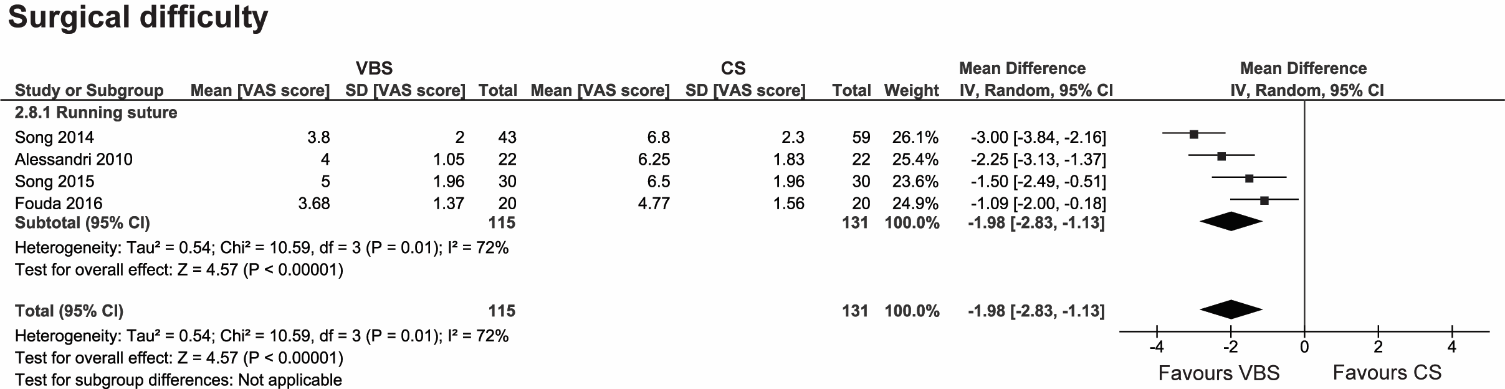


#### **Fig. S9**

Funnel plots of outcomes and subgroup analysis according to surgery type for the use of VBS versus CS in OB/GYN surgery


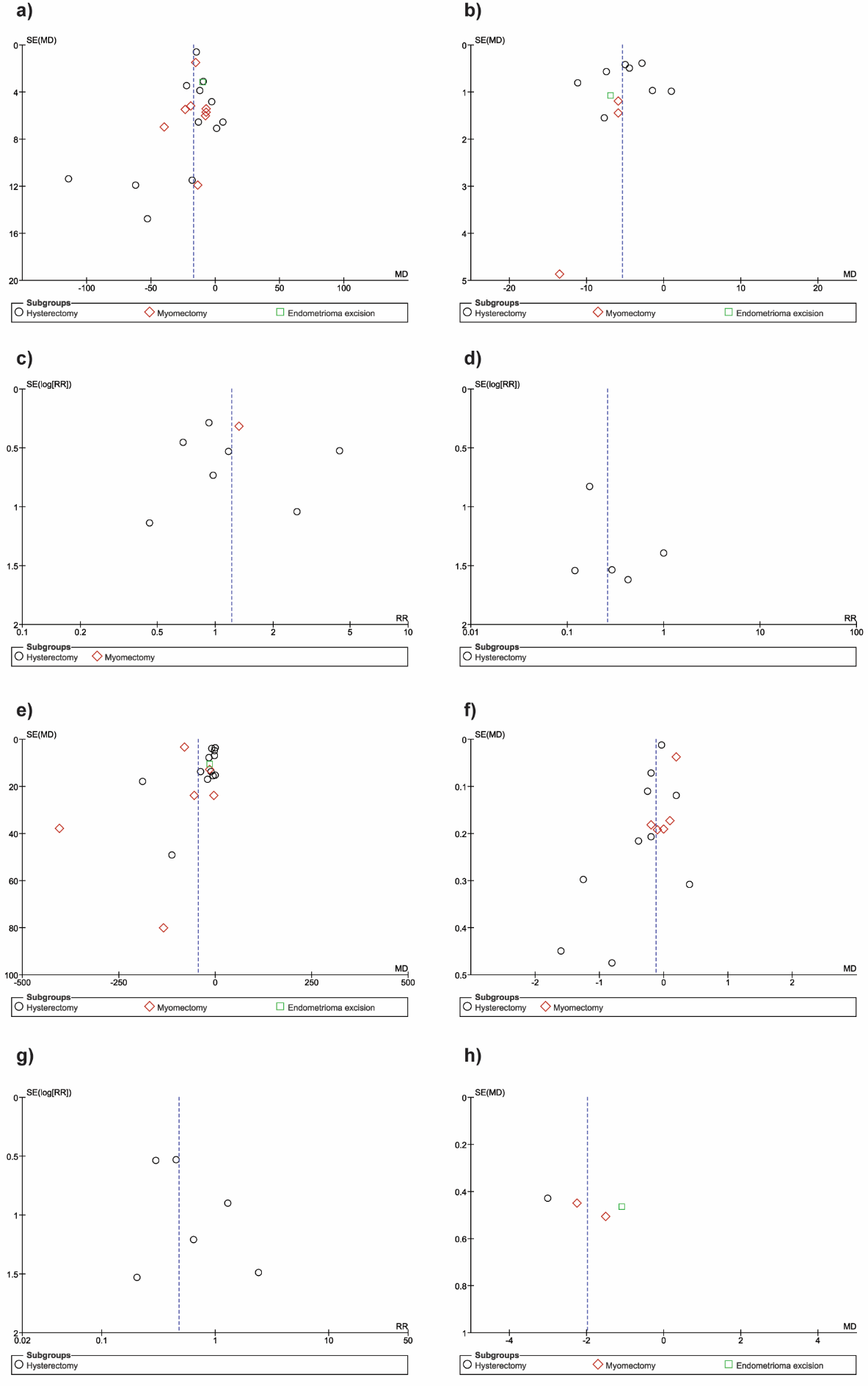


## Supplementary tables

#### Table S 1

Characteristics of the included comparative studies comparing VBS to CS in OB/GYN procedures. Risk of bias was assessed using the Downs and Black checklist for quality assessment for RCTs and non-RCTs [23]

| **Study ID** | **Study design** | **Country** | **Surgery type** | **Inclusion criteria** | **Exclusion criteria** | **Sample size** | | **Intervention (VBS)** | **Comparator** | | **Risk of bias** | |
| --- | --- | --- | --- | --- | --- | --- | --- | --- | --- | --- | --- | --- |
|  |  |  |  |  |  | **V-Loc** | **Comparator** |  | **Suture (CS)** | **Type** | **Score** | **Rating** |
| Alessandri 2010 [9] | RCT | Italy | Laparoscopic myomectomy | Up to 3 intramural myomas with free margin, reproductive age, largest myoma diameter ≤10 cm | Previous uterine surgery, diseases requiring additional surgical treatment, BMI >29kg/m^2^, contraindications for general anesthesia, psychiatric disorders precluding informed consent | 22 | 22 | V-Loc 180 | Vicryl | RS | 19 | Fair |
| Angioli 2012 [40] | Prospective study group with retrospective control group | Italy | Laparoscopic myomectomy | Single symptomatic myoma with free margin, reproductive age >18 years, largest myoma 3-5 cm in diameter, absence of other gynecologic pathology, hemoglobin level >12, WHO performance status <1 | Subserosal, sub mucosal, posterior, or intraligamentous myoma, previous pelvic surgery, gynecologic neoplasms, psychiatric disorders precluding informed consent, coagulation defects, anticoagulant therapy, liver disease, previous treatment with gonadotropin-releasing hormone analog, contraindications for general anesthesia, BMI >35, pelvic inflammatory disease, menopausal status | 19 | 20 | 2-0 V-Loc 90 | 0-polyglactin | RS | 16 | Fair |
| Aoki 2014 [41] | Retrospective | Japan | Laparoscopic myomectomy | ≥1 intramural myoma | Concomitant procedure or adhesiolysis for severe adhesions | 41 | 42 | 0 V-Loc 180 | 0-polysorb | RS | 16 | Fair |
| Arena 2021 [42] | Retrospective | Italy | Laparoscopic myomectomy | Age >18 years, laparoscopic myomectomy as primary surgical indication | Laparotomic conversion, no need for myometrial suturing, use of multiple different sutures to close the uterine defect | 83 | 81 | 0 V-Loc | 1/0 Biosyn absorbable monofilament | RS | 17 | Fair |
| Bassi 2013 [43] | Retrospective | Canada | Laparoscopic hysterectomy | TLH | Laparotomy, laparoscopic subtotal hysterectomy, robot-assisted laparoscopy, additional surgical procedures, incomplete medical file | 63 | 139 | V-Loc | 0-polydioxanone | IS | 11 | Poor |
| Brown 2016 [32] | Retrospective | USA | Laparoscopic or robot-assisted hysterectomy | Laparoscopic or robot-assisted hysterectomy | Conversion to open procedure, laparoscopic-assisted vaginal hysterectomy | 375 | 99 | V-Loc 180 | Vicryl polyglactin 910 | RS | 14 | Poor |
| Cong 2016 [28] | Retrospective | China | Laparoscopic hysterectomy | TLH | Supracervical hysterectomy, cancer surgery, concomitant urogynecological procedure | 306 | 184 | V-Loc 180 | CL-914 polysorb | CS | 13 | Poor |
| Fouda 2016 [5] | RCT | Egypt | Laparoscopic excision of ovarian endometrioma | Women of reproductive age, unilateral ovarian endometrioma | Age <20 or >42 years, endometrioma mean diameter <3 cm or >10 cm, severe pelvic adhesions, uterine myoma requiring excision, previous ovarian cystectomy or myomectomy, use of hormonal treatment within 4 months before surgery, pelvic inflammatory disease, coagulation defects, pregnancy, infertile patients with AMH level <2 ng/ml, compromised cardiopulmonary status, contraindications for general anesthesia | 20 | 20 | 2-0 V-Loc 180 | 2-0 polyglactin 910 vicryl | RS | 23 | Good |
| Huang 2013 [27] | Prospective | Taiwan | Mini-laparotomy myomectomy | Not reported | Not reported | 34 | 34 | V-Loc | 1-0 Safil | IS & RS | 12 | Poor |
| Karacan 2018 [39] | Retrospective | Turkey | Laparoscopic hysterectomy | TLH due to benign diseases, intracorporeal vaginal cuff closure | Suspicion of malignancy, potential need for surgical prolapse repair, transvaginal or extracorporeal vaginal cuff closure | 208 | 89 | V-Loc 180 | Running 1-0 Safil (serosa)" | IS | 15 | Fair |
| Khoiwal 2021 [44] | Prospective | India | Laparoscopic hysterectomy | TLH for benign uterine pathology, able to communicate by phone and answer questions, fit to withstand surgery | (Pre)malignant disease of uterus, cervix, or ovaries, adnexal mass, pregnancy, genital prolapse, coagulation disorders, contraindications for laparoscopy | 44 | 65 | V-Loc 180 | Polyglactin 910 vicryl | RS | 20 | Good |
| Kim 2016 [8] | Retrospective | South Korea | Laparoscopic hysterectomy | TLH for benign conditions | Not reported | 106 | 64 | 0 V-Loc | Polyglactin 910 vicryl | RS | 16 | Fair |
| Kim 2018 [45] | Retrospective | South Korea | Laparoscopic hysterectomy | TLH for benign conditions | Suspicion of malignancy, coagulation disorder, simultaneous surgical interventions | 122 | 212 | V-Loc 90 | 1-0 Vicryl | RS | 15 | Fair |
| Kumakiri 2020 [46] | Retrospective | Japan | Laparoscopic myomectomy | Laparoscopic myomectomy | Concomitant surgery that might influence post-operative adhesion formation | 28 | 187 | 0-V-loc 180 | 1-0 polyglactin -10 vicryl | RS | 17 | Fair |
| Lee 2017 [47] | Prospective | South Korea | Laparoscopic hysterectomy | Age ≥ 20 years, TLH, no gynecological malignancy, fit for laparoscopic surgery, uterine size ≤ 1,000 g | Uterine size > 1,000 gm suspected gynecological malignancy, >3 prior laparotomies | 37 | 48 | 2-0 V-Loc 90 | 0-Polysorb | RS | 17 | Fair |
| López 2019 [29] | RCT | Colombia | Laparoscopic hysterectomy | Age >18 years, TLH | Risk factors for vaginal cuff dehiscence | 75 | 75 | V-Loc 90 | Glyconate monofilament | RS | 22 | Good |
| Morgan-Ortiz 2013 [30] | Prospective | Mexico | Laparoscopic hysterectomy | Not reported | Not reported | 25 | 25 | 0-V-Loc 90 | Polyglactin 910 vicryl | RS | 19 | Fair |
| Nawfal 2012 [48] | Retrospective | USA | Robot-assisted hysterectomy | Scheduled robot-assisted TLH | Supracervical hysterectomy, cancer surgery, concomitant urogynecological procedure | 69 | 133 | V-Loc 180 | 1-0 polyglactin 910 | IS | 16 | Fair |
| Neubauer 2013 [36] | Retrospective | USA | Robot-assisted hysterectomy | Robot-assisted TLH | Not reported | 76 | 58 | 2-0 V-Loc 180 | Polyglactin 910 vicryl | RS | 10 | Poor |
| Song 2014 [49] | Prospective | South Korea | Laparoscopic hysterectomy | Gynecologic disease as indication for hysterectomy, no gynecologic malignancy, fit for laparoscopic surgery | Age ≤18 years, uterine size ≥20 gestational weeks, recent cancer diagnosis, patient inability to understand and provide written informed consent | 43 | 59 | 1-0 V-Loc 180 | 2-0 polydioxanone | RS | 15 | Fair |
| Song 2015 [31] | Prospective | South Korea | Laparoscopic myomectomy | Myomas causing symptoms, planned laparoscopic myomectomy, ≤3 myomas, largest myoma ≤12 cm, not pregnant, age between 18 and 55 years | Dominant pedunculated subserosal or submucosal myoma, concomitant complex surgery, malignant uterine or adnexal diseases, medical comorbidities or psychiatric disorders affecting follow-up and/or compliance, refusal to participate or give consent | 30 | 30 | 1-0 V-Loc 180 | Monosyn or 1-0 Vicryl | RS | 15 | Fair |
| Tillmanns 2016 [37] | RCT | USA | Robot-assisted hysterectomy | All indications for robotic hysterectomy | Anticipated need for post-operative pelvic radiation therapy | 33 | 34 | 2-0 V-Loc 180 | 1-0 Vicryl | IS | 19 | Fair |
| Tinelli 2016 [50] | Retrospective | Italy | Laparoscopic myomectomy | Laparoscopic myomectomy, myoma size ≤9 cm, ≤3 symptomatic subserous or intramural myomas | >3 myomas, myoma size >9 cm preoperative hemoglobin level <85 g/l, cardiovascular disease, pulmonary obstructive disease, pelvic or abdominal radiation therapy, severe hip disease, poor bone barrow, renal, or hepatic function, submucosal myomas, ovarian or uterine neoplastic lesions, metastases beyond the uterus, contraindication for general anesthesia, systemic infection, abnormal cervical smear, pregnancy | 360 | 360 | V-Loc | 2-0 Vicryl | IS | 15 | Fair |
| Tsafrir 2017 [26] | RCT | USA | Robot-assisted hysterectomy | Robot-assisted TLH | Non-benign indications for TLH, concomitant urogynecological procedures | 30 | 30 | V-Loc 90 | 0 GS-21 Polysorb | RS | 22 | Good |
| Zhou 2014 [51] | Retrospective | USA | Robot-assisted hysterectomy | Robot-assisted TLH for benign indications | Incomplete follow-up records, conversion to abdominal hysterectomy, supracervical hysterectomy, malignant specimens, cases where Quill barbed sutures were used for vaginal cuff closure | 49 | 44 | V-Loc 180 | 0 Vicryl with Lapra-Ty | IS | 13 | Poor |

BMI – Body mass index, CS – Conventional suture, IS – Interrupted suture, RS – Running suture, RCT – Randomized controlled trial, TLH – Total laparoscopic hysterectomy

#### Table S2

Detailed risk of bias assessment according to the modified Downs and Black checklist for quality assessment

| **Study ID** | **Reporting bias (max 10 points)** | **External validity (max 3 points)** | **Internal validity (bias) (max 7 points)** | **Internal validity (confounding) (max 7 points)** | **Total score (max 27 pounts)** | **Bias rating** |
| --- | --- | --- | --- | --- | --- | --- |
|  |  |  |  |  |  |  |
| Alessandri 2010 [9] | 8 | 2 | 4 | 5 | 19 | Fair |
| Angioli 2012 [40] | 9 | 1 | 4 | 2 | 16 | Fair |
| Aoki 2014 [41] | 9 | 1 | 4 | 2 | 16 | Fair |
| Arena 2021 [42] | 9 | 1 | 4 | 3 | 17 | Fair |
| Bassi 2013 [43] | 6 | 1 | 3 | 1 | 11 | Poor |
| Brown 2016 [32] | 9 | 1 | 3 | 1 | 14 | Poor |
| Cong 2016 [28] | 7 | 2 | 3 | 1 | 13 | Poor |
| Fouda 2016 [5] | 10 | 1 | 5 | 7 | 23 | Good |
| Huang 2013 [27] | 7 | 1 | 3 | 1 | 12 | Poor |
| Karacan 2018 [39] | 8 | 1 | 3 | 3 | 15 | Fair |
| Khoiwal 2021 [44] | 10 | 2 | 5 | 3 | 20 | Good |
| Kim 2016 [8] | 9 | 1 | 4 | 2 | 16 | Fair |
| Kim 2018 [45] | 9 | 1 | 3 | 2 | 15 | Fair |
| Kumakiri 2020 [46] | 9 | 1 | 3 | 4 | 17 | Fair |
| Lee 2017 [47] | 10 | 2 | 4 | 1 | 17 | Fair |
| López 2019 [29] | 9 | 1 | 6 | 6 | 22 | Good |
| Morgan-Ortiz 2013 [30] | 9 | 1 | 5 | 4 | 19 | Fair |
| Nawfal 2012 [48] | 9 | 1 | 3 | 3 | 16 | Fair |
| Neubauer 2013 [36] | 3 | 2 | 2 | 3 | 10 | Poor |
| Song 2014 [49] | 9 | 1 | 3 | 2 | 15 | Fair |
| Song 2015 [31] | 8 | 1 | 4 | 2 | 15 | Fair |
| Tillmanns 2016 [37] | 7 | 1 | 5 | 6 | 19 | Fair |
| Tinelli 2016 [50] | 8 | 2 | 3 | 2 | 15 | Fair |
| Tsafrir 2017 [26] | 10 | 1 | 5 | 6 | 22 | Good |
| Zhou 2014 [51] | 8 | 1 | 3 | 1 | 13 | Poor |
